# Bayesian Inference for the Kumaraswamy Distribution under Generalized Progressive Hybrid Censoring

**DOI:** 10.3390/e22091032

**Published:** 2020-09-15

**Authors:** Jiayi Tu, Wenhao Gui

**Affiliations:** Department of Mathematics, Beijing Jiaotong University, Beijing 100044, China; 17271132@bjtu.edu.cn

**Keywords:** Kumaraswamy distribution, generalized progressive hybrid censoring, maximum liklihood estimation, bayesian estimation, Lindley’s approximation, Tierney and Kadane method

## Abstract

Incomplete data are unavoidable for survival analysis as well as life testing, so more and more researchers are beginning to study censoring data. This paper discusses and considers the estimation of unknown parameters featured by the Kumaraswamy distribution on the condition of generalized progressive hybrid censoring scheme. Estimation of reliability is also considered in this paper. To begin with, the maximum likelihood estimators are derived. In addition, Bayesian estimators under not only symmetric but also asymmetric loss functions, like general entropy, squared error as well as linex loss function, are also offered. Since the Bayesian estimates fail to be of explicit computation, Lindley approximation, as well as the Tierney and Kadane method, is employed to obtain the Bayesian estimates. A simulation research is conducted for the comparison of the effectiveness of the proposed estimators. A real-life example is employed for illustration.

## 1. Introduction

### 1.1. Kumaraswamy Distribution

Given that the classical probability distribution functions like beta, normal, log-normal, Student-*t* (Contreras-Reyes et al. [1]) and other empirical distributions cannot fit hydrological data quite well, Kumaraswamy [2] came up with a new two-parameter distribution to be specifically applicable for hydrological problems. The cumulative distribution function (cdf) of the Kumaraswamy distribution is

(1) $$\begin{array}{c} F\left(x\right)=1-{\left(1-x^{\alpha }\right)}^{\beta },\;0\leq x\leq 1, \end{array}$$

where both α and β represent the positive shape parameters of the distribution, which is denoted by *K*(α,β) in this paper. The following presents the corresponding probability density function (pdf):

(2) $$\begin{array}{c} f\left(x\right)=\alpha \beta x^{\alpha -1}{\left(1-x^{\alpha }\right)}^{\beta -1},\;0\leq x\leq 1. \end{array}$$

In addition, an in-depth observation is made on the reliability function of the Kumaraswamy distribution, which is shown below:

(3) $$R\left(t\right)={\left(1-t^{\alpha }\right)}^{\beta },\;t>0.$$

**Figure 1:**
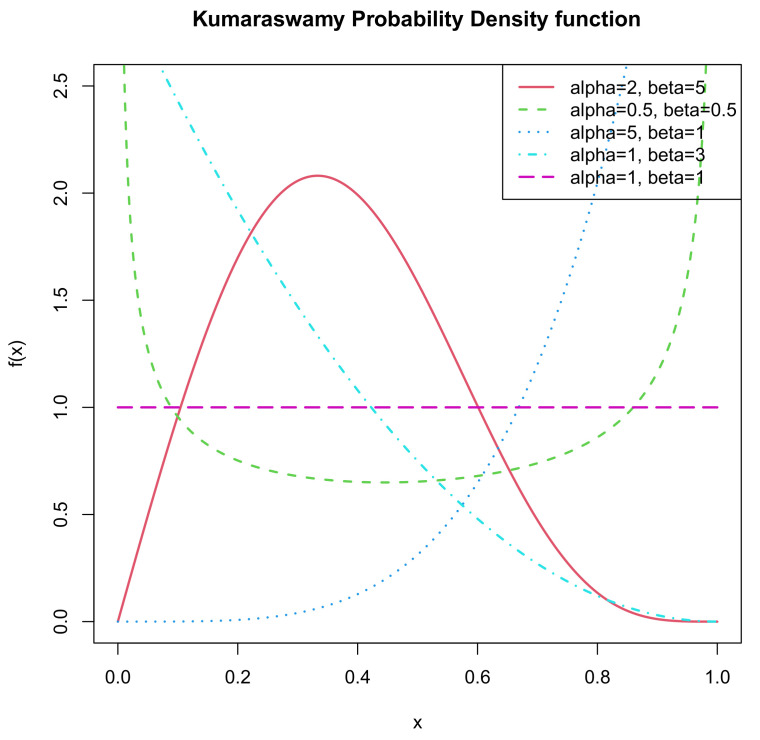
pdf of *K*(α,β).

**Figure 2:**
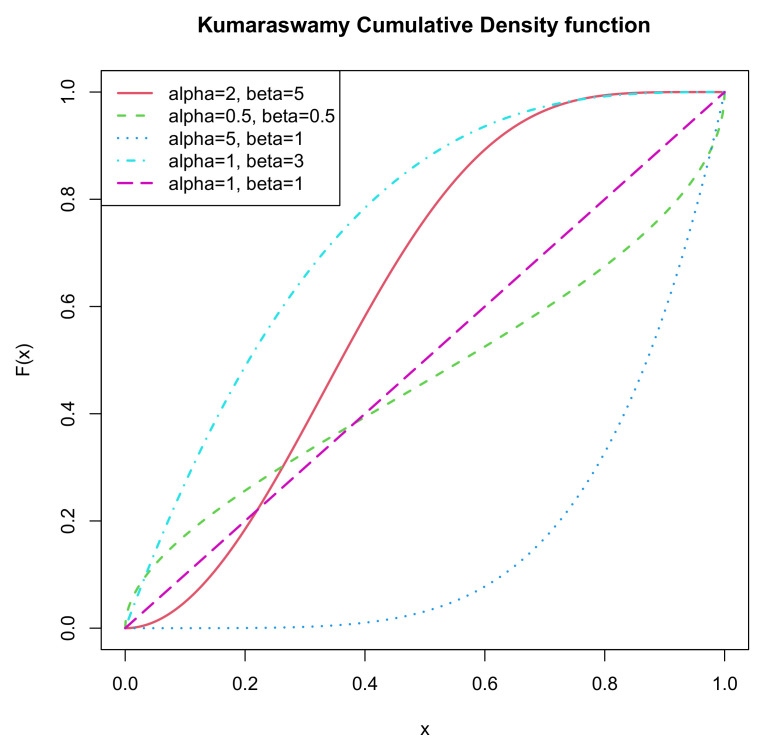
cdf of *K*(α,β).

An increasing number of statisticians have started to study it since it has many flexible shape properties which are identical with the beta distribution. In light of different values of parameters, the pdf of the Kumaraswamy distribution can appear to have diverse shapes. It is uniantimodal if α,β<1; it is unimodal if α,β>1; it is decreasing if α≤1,β>1; it is increasing if α>1,β≤1; and it is constant if α=β=1. Figure 1 and Figure 2 illustrate the pdf and cdf respectively for the above five cases, namely α=2,0.5,5,1,1 and β=5,0.5,1,3,1. One may refer to Mitnik [3] for more information. Futher, assigning different value combinations of (α,β), we can convert the Kumaraswamy distribution into some other distributions, like uniform, exponential, beta.

In comparison, the Kumaraswamy distribution is superior to the beta distribution because the cdf of the beta distribution contains a form of integral which cannot be simplified. However, the cdf of the Kumaraswamy distribution is in explicit expression, which results in some advantages of tractability.

What is more, the Kumaraswamy distribution fits well with some natural phenomena, such as daily rainfall, water flows and other pertinent fields, see Fletcher and Ponnambalam [4], Sundar and Subbiah [5], Ponnambalam et al. [6] as well as Seifi et al. [7], especially for the outcomes of which possess upper and lower bounds, like the heights of people, test scores, air temperatures, economic data, etc. Estimation for the Kumaraswamy distribution has gradually attracted the attention of scholars in recent years. Lemonte [8] obtained modified maximum likelihood estimators which are unbiased in the second stage. Based on the bias correction estimations, they studied a bias-corrected method using parametric bootstrap. Then, statisticians attempted to study the Kumaraswamy distribution under different censoring schemes and started to combine classical and Bayesian methods. Ghosh and Nadarajah [9] discussed Bayesian estimation using two loss functions under three types of censoring schemes: left censoring, single type-II censoring and double type-II censoring with one parameter known. Sultana et al. [10] discussed and estimated parameters of the Kumaraswamy distribution with hybrid censoring scheme, and recently Sultana et al. [11] combined hybrid with progressive type I censoring schemes to explore the parameter estimation problems for the same distribution.

### 1.2. Generalized Progressive Hybrid Censoring Scheme

Life testing experiments are widely used in engineering, biology, machinery and other fields of study, which can be summarized as mathematical and probabilistic models of survival analysis. In reality, several restrictions, such as time and cost, prevent us from observing the failure time of all units. It is common to cease in the middle of process before all the observations fail. Such limitations result in censored data. Among all censoring cases, the two most typical schemes are Type-I as well as Type-II censoring schemes. According to previous literature, plenty of authors have discussed this aspect and one may consult Meeker and Escobar [12] which includes methods of handling Type-I as well as Type-II censored data.

An accidental pause or an unavoidable loss of the experiment units is likely to happen before the final termination. However, the constraint in those two censoring schemes is that removal cannot occur to the units in the duration of the experiment. To resolve this inflexibility, Cohen [13] first introduced progressive censoring scheme. A progressive Type-II censoring sample will be given below. Assuming that *n* independent units of a common lifetime distribution denoted by $X_{1},X_{2},\ldots ,X_{n}$ are put in the experiment at *t* = 0, when the first failure occurs, among n−1 survivals, we take $R_{1}$ units out of the experiment at random. Similarly, when the second failure happens, we randomly remove $R_{2}$ units among the $n-2-R_{1}$ survivals. We conduct the repeated procedure till the observation of the mth failure. On the occasion of the *m*-th failure, removal occurs to the $R_{m}=n-m-R_{1}-R_{2}-\ldots -R_{m-1}$ survivals. During the experiment, the progressive censoring scheme ($R_{1},R_{2},\ldots ,R_{m}$), which is considered to be progressive type-II censored scheme, is prefixed satisfying $\sum_{i=1}^{m}R_{i}+m=n$. *m* ordered failure times are written in light of $X_{1}<X_{2}<\ldots <X_{m}$.

According to weakness harbored by the progressive type-II censored scheme, if the experimental units are highly reliable, this experiment will last quite long. Hence, the progressively hybrid censoring scheme was introduced by Kundu and Joarder [14]. For the censoring scheme, the implementation of *n* independent identical distributed units is used. The experimenter will cease the operation at $\min\left\{T,X_{m}\right\}$. Here, the time *T* as well as 1≤m≤n is determined ahead of time. In the context of the progressive type-II censored scheme, the span of the experiment will not take a longer duration than *T*.

**Figure 3:**
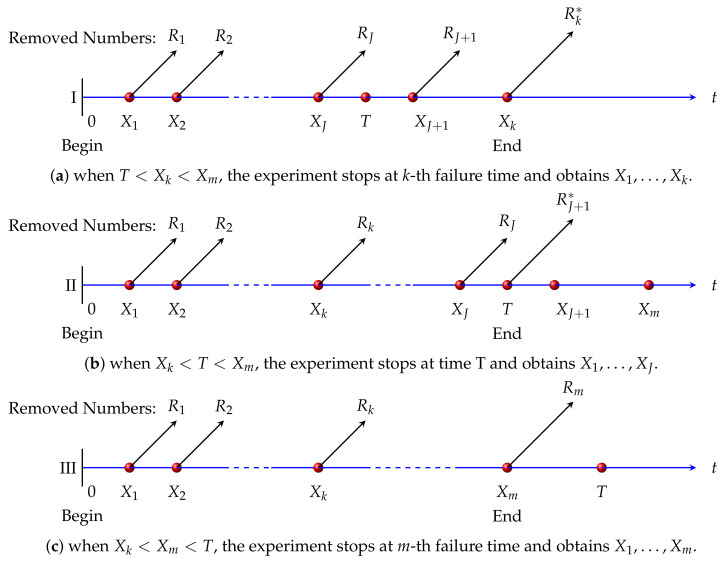
Generalized progressive hybrid censoring scheme.

However, given that when the prefixed termination time *T* may be small, the observation we obtained would be insufficient. Therefore, a new mode of censoring scheme—generalized progressive hybrid censoring scheme—is proposed by Cho and Sun [15], which enables us to obtain a predetermined series of failures. How to get generalized progressive hybrid censored data is described below by a graphic illustration in Figure 3.

Assume that our research group possesses *n* independent units of a common lifetime distribution. The corresponding lifetime is denoted by $X_{1},X_{2},\ldots ,X_{n}$. The integers *k* and *m*(k<m) have been under predetermination between zero and n as well as $R_{1},R_{2},\ldots ,R_{m}$ which can satisfy the equation $\sum_{i=1}^{m}R_{i}+m=n$ function as preplanned integers. On the arrival of the first failure $X_{1}$, we randomly remove $R_{1}$ units. When the second failure $X_{2}$ happens, we take random removal of $R_{2}$ units out of the $n-2-R_{1}$ survivals. The process is repeated and terminated at $T^{*}=\max\left\{\min\left\{T,X_{m}\right\},X_{k}\right\}$ with the rest of the survival units under the removal. It greatly modified the previous schemes so that we can choose to continue the experiment when the sample is insufficient at the prefixed cut-off time *T*. On the condition of the generalized progressive hybrid censoring scheme, researchers would like to obtain *m* failures, while they can also adopt *k* failures which are regarded as the bare minimum. We denote the generalized progressive hybrid censoring scheme as ($R_{1},R_{2},\ldots ,R_{m}$). Let *J* be the observed failure times before arriving at the predetermined time *T*. The generalized progressive censoring scheme can be classified into these cases as below:

$$\begin{array}{cc} \mathrm{Case}\;I & :\;X_{1},\ldots ,X_{J},\ldots ,X_{k},\;\mathrm{for}\;T<X_{k}<X_{m},\\ \mathrm{Case}\;\mathrm{II} & :\;X_{1},\ldots ,X_{k},\ldots ,X_{J},\;\mathrm{for}\;X_{k}<T<X_{m},\\ \mathrm{Case}\;\mathrm{III} & :\;X_{1},\ldots ,X_{k},\ldots ,X_{m},\;\mathrm{for}\;X_{k}<X_{m}<T. \end{array}$$

Case III represents the progressive Type-II censoring scheme and the mixture of Case II and Case III is the progressive hybrid censoring scheme. Hence, evidently, Case I is the modification of this scheme. Let $R_{1}=0,R_{2}=0,\ldots ,R_{m}=0$, we can obtain complete sample. We also assume $R_{1}=\ldots =R_{m-1}=0,R_{m}=n-m$, in order to get the type-II censored sample.

According to the generalized progressive hybrid censoring scheme, the likelihood equations of the three cases are

(4) $$\begin{array}{cc} \mathrm{Case}\;I & :\;L_{1}=Q_{1}\prod_{j=1}^{k-1}f\left(x_{j}\right){\left[1-F(x_{j})\right]}^{R_{j}}f\left(x_{k}\right){\left[1-F(x_{k})\right]}^{R_{k}^{*}},\\ \mathrm{Case}\;\mathrm{II} & :\;L_{2}=Q_{2}\prod_{j=1}^{J}f\left(x_{j}\right){\left[1-F(x_{j})\right]}^{R_{j}}{\left[1-F(T)\right]}^{R_{J+1}^{*}},\\ \mathrm{Case}\;\mathrm{III} & :\;L_{3}=Q_{3}\prod_{j=1}^{m}f\left(x_{j}\right){\left[1-F(x_{j})\right]}^{R_{j}}, \end{array}$$

where $Q_{1}=\left[\prod_{j=1}^{k}\sum_{k=j}^{m}\left(R_{k}+1\right)\right]$, $Q_{2}=\left[\prod_{j=1}^{J}\sum_{k=j}^{m}\left(R_{k}+1\right)\right]$, $Q_{3}=\left[\prod_{j=1}^{m}\sum_{k=j}^{m}\left(R_{k}+1\right)\right]$, $R_{k}^{*}=n-k-\sum_{i=1}^{k-1}R_{i}=R_{k}$ and $R_{J+1}^{*}=n-J-\sum_{i=1}^{J}R_{i}$.

As far as we know, estimations for the Kumaraswamy distribution under generalized progressive hybrid censoring scheme have not been done yet in previous literature. Hence, this paper will discuss the estimations for parameters and reliability of the Kumaraswamy distribution on the basis of this model.

The rest of the article consists of the following parts. In the next section, the maximum likelihood estimators of the two unknown parameters as well as reliability function of the model will be derived theoretically. For all unknown quantities, Bayes estimators are achieved in Section 3 under three diverse loss functions employing Lindley’s approximation. Then in Section 4, a simulation experiment will be carried out in light of the conclusion in Section 2 and Section 3. Data analysis is demonstrated in Section 5. Finally, Section 6 concludes the thesis.

## 2. Maximum Likelihood Estimation

In dealing with reliability problems and survival analysis, an effective and classical approach widely employed by statisticians is maximum likelihood estimation (MLE). By employing this method, two unknown parameters will be derived. As a result, MLE of R(t) will also be acquired. Plugging the pdf and cdf of the Kumaraswamy distribution, i.e., (2) and (1), into the likelihood Formula (4), the likelihood functions of α and β after neglecting the constants are expressed as

$$\begin{array}{cc} \mathrm{Case}\;I:\;L_{1}\propto & {\left(\alpha \beta \right)}^{k}\prod_{j=1}^{k}x_{j}^{\alpha -1}{\left(1-x_{j}^{\alpha }\right)}^{\beta (1+R_{j})-1},\\ \mathrm{Case}\;\mathrm{II}:\;L_{2}\propto & {\left(\alpha \beta \right)}^{J}{\left(1-T^{\alpha }\right)}^{\beta R_{J+1}^{*}}\prod_{j=1}^{J}x_{j}^{\alpha -1}{\left(1-x_{j}^{\alpha }\right)}^{\beta (1+R_{j})-1},\\ \mathrm{Case}\;\mathrm{III}:\;L_{3}\propto & {\left(\alpha \beta \right)}^{m}\prod_{j=1}^{m}x_{j}^{\alpha -1}{\left(1-x_{j}^{\alpha }\right)}^{\beta (1+R_{j})-1}. \end{array}$$

Disregarding the constant, the log-likelihood functions are

Case I:

$$\begin{array}{cc} l_{1}\;\;\propto & k\log\alpha \beta +\left(\alpha -1\right)\sum_{j=1}^{k}\log x_{j}+\beta \left[\sum_{j=1}^{k}\left(1+R_{j}\right)\log\left(1-x_{j}^{\alpha }\right)\right]-\sum_{j=1}^{k}\log\left(1-x_{j}^{\alpha }\right), \end{array}$$

Case II:

$$\begin{array}{cc} l_{2}\;\propto & J\log\alpha \beta +\left(\alpha -1\right)\sum_{j=1}^{J}\log x_{j}+\beta \left[\sum_{j=1}^{J}\left(1+R_{j}\right)\log\left(1-x_{j}^{\alpha }\right)+R_{J+1}^{*}\log\left(1-T^{\alpha }\right)\right]\\ \; & -\sum_{j=1}^{J}\log\left(1-x_{j}^{\alpha }\right), \end{array}$$

Case III:

$$\begin{array}{cc} l_{3}\;\;\propto & m\log\alpha \beta +\left(\alpha -1\right)\sum_{j=1}^{m}\log x_{j}+\beta \left[\sum_{j=1}^{m}\left(1+R_{j}\right)\log\left(1-x_{j}^{\alpha }\right)\right]-\sum_{j=1}^{m}\log\left(1-x_{j}^{\alpha }\right). \end{array}$$

In order to simplify the above expressions, we combine Case I, II and III and obtain the log-likelihood function as

(5) $$\begin{array}{cc} l\;\;\propto & D\log\alpha \beta +\left(\alpha -1\right)\sum_{j=1}^{D}\log x_{j}+\beta \left[\sum_{j=1}^{D}\left(1+R_{j}\right)\log\left(1-x_{j}^{\alpha }\right)+E\left(\alpha \right)\right]\\ \; & -\sum_{j=1}^{D}\log\left(1-x_{j}^{\alpha }\right), \end{array}$$

where for Case I, D=k, E(α)=0; for Case II, D=J, $E\left(\alpha \right)=R_{J+1}^{*}\log\left(1-T^{\alpha }\right)$; for Case III, D=m, E(α)=0.

We take the partial derivatives of the above function (5) for α and β respectively and get a set of likelihood equations as follows:

(6) $$\frac{\partial l}{\partial \beta }=\frac{D}{\beta }+\sum_{j=1}^{D}\left(1+R_{j}\right)\log\left(1-x_{j}^{\alpha }\right)+E\left(\alpha \right)=0,$$

and

(7) $$\frac{\partial l}{\partial \alpha }=\frac{D}{\alpha }+\sum_{j=1}^{D}\log x_{j}+\beta \left[\sum_{j=1}^{D}\left(1+R_{j}\right)\frac{-x_{j}^{\alpha }\log x_{j}}{1-x_{j}^{\alpha }}+E^{\left(1\right)}\left(\alpha \right)\right]+\sum_{j=1}^{D}\frac{x_{j}^{\alpha }\log x_{j}}{1-x_{j}^{\alpha }},$$

where

$$E^{\left(1\right)}\left(\alpha \right)=\left\{\begin{array}{cc} 0 & \mathrm{for}\;\mathrm{Case}\;I\;\mathrm{and}\;\mathrm{III},\\ -R_{J+1}^{*}\frac{T^{\alpha }\log T}{1-T^{\alpha }} & \mathrm{for}\;\mathrm{Case}\;\mathrm{II}. \end{array}\right.$$

By solving the roots of the above set of equations, the MLEs of two parameters are able to be attained theoretically. From the Equation (6), we gain the maximum likelihood estimate of β as

(8) $$\hat{\beta }\left(\alpha \right)=-\frac{D}{\sum_{j=1}^{D}\left(1+R_{j}\right)\log\left(1-x_{j}^{\alpha }\right)+E\left(\alpha \right)}.$$

Placing the estimation value of β into the Equation (7), we can get

(9) g(α)=α,

where

$$g\left(\alpha \right)=-\frac{D}{\sum_{j=1}^{D}\log x_{j}+\hat{\beta }\left(\alpha \right)\left[\sum_{j=1}^{D}\left(1+R_{j}\right)\frac{-x_{j}^{\alpha }\log x_{j}}{1-x_{j}^{\alpha }}+E^{\left(1\right)}\left(\alpha \right)\right]+\sum_{j=1}^{D}\frac{x_{j}^{\alpha }\log x_{j}}{1-x_{j}^{\alpha }}}.$$

Obviously, it is hard to simplify and gain solutions of closed forms, since both (8) and (9) are nonlinear. In this situation, updating the estimates seems to be an effective method to gain approximate solution of α. This iterative algorithm has been proposed by Kundu [16]. Here, we give a brief description. Begin with an initial assumption of α, noted by $\alpha^{\left(0\right)}$, then gain $\alpha^{\left(1\right)}=g\left(\alpha^{\left(0\right)}\right)$ and repeat this iteration and we gain $\alpha \left(n+1\right)=g(\alpha^{\left(n\right)})$. When the precision meet the tolerance limit which is set beforehand $|\alpha^{\left(n+1\right)}-\alpha^{\left(n\right)}|<\varepsilon$, we stop the iterative process. Once we get the MLE of α, denoted by $\hat{\alpha }$, the MLE of β is deduced as $\hat{\beta }=\hat{\beta }\left(\hat{\alpha }\right)$. As long as the MLEs are obtained, we can substitute these two estimates into (3) to gain the MLE of R(t) as:

(10) $$\hat{R}\left(t\right)={\left(1-t^{\hat{\alpha }}\right)}^{\hat{\beta }},\;t>0.$$

## 3. Bayesian Estimation

Bayesian estimation which considers prior information as well as sample information is a fresh but efficient approach in comparison with MLE. This more comprehensive estimation method is usually more precise than the maximum likelihood estimation. In this part, Bayesian estimation of the model parameters α, β and reliability function will be discussed.

### 3.1. Symmetric and Asymetric Loss Functions

In statistics, we usually estimate a parameter by minimizing the loss function. There are lots of diverse symmetric or asymmetric loss functions. Here we will interpret the three typical loss functions which are taken into consideration. In all of the following cases, d(η) stands for the true value of the unknown parameter and $\hat{d}\left(\eta \right)$ is the corresponding estimate of d(η). The symmetric one refers to the squared error loss (SEL) function. It is the most prevalent one and can be easily proved to be right based on minimum variance-unbiased estimation. The definition and corresponding Bayes estimator are

$$L_{S}\left[d\left(\eta \right),\hat{d}\left(\eta \right)\right]={\left[\hat{d}\left(\eta \right)-d\left(\eta \right)\right]}^{2},$$

$${\hat{d}}_{SEL}=E_{\eta }\left(\eta |\underline{x}\right).$$

However, due to symmetry, the overestimation of SEL function has equal weight as underestimation of the same magnitude, which gives rise to the emergence of a large number of asymmetric functions. The Linex loss (LL) function [17], an extensively adopted asymmetric loss function, is another loss function discussed in this paper. The definition and corresponding Bayes estimator are

$$L_{L}\left[d\left(\eta \right),\hat{d}\left(\eta \right)\right]=e^{p\left[\hat{d}\left(\eta \right)-d\left(\eta \right)\right]}-p\left[\hat{d}\left(\eta \right)-d\left(\eta \right)\right]-1,\;p\neq 0,$$

$${\hat{d}}_{LL}=-\frac{1}{p}\log\left[E_{\eta }\left(e^{-p\eta }|\underline{x}\right)\right].$$

The parameter *p* represents the deviation direction, and the degree of deviation is reflected by its magnitude. When p<0, the underestimation is greater than the overestimation and the opposite is the case when p>0. When parameter *p* converges towards zero, the linex loss function can be converted to SEL loss function.

In addition, an asymmetric loss function—the general entropy loss (EL) function is also considered whose definition and corresponding Bayes estimator are

$$L_{E}\left[d\left(\eta \right),\hat{d}\left(\eta \right)\right]={\left[\frac{\hat{d}\left(\eta \right)}{d(\eta )}\right]}^{q}-q\log\frac{\hat{d}\left(\eta \right)}{d(\eta )}-1,\;q\neq 0,$$

$${\hat{d}}_{EL}={\left[E_{\eta }\left(\eta^{-q}|\underline{x}\right)\right]}^{-\frac{1}{q}}.$$

Here, the positive error is greater than the negative one when q>0, and the opposite is the case when q>0.

### 3.2. Prior and Posterior Distributions

Since a natural conjugate bivariate prior distribution for α and β does not exist, we employ the same assumption as Kundu and Pradhan [18], supposing that α and β are subjected to gamma distributions independently for the reason that gamma distribution can be adapted to various shapes depending on parameter values. Now, the joint prior distribution, ignoring the constant coefficient, is in the form of

(11) $$\pi \left(\alpha ,\beta \right)\;\alpha^{a-1}\beta^{c-1}e^{-b\alpha }e^{-d\beta },\;\alpha >0,\beta >0.$$

Here, the positive hyperparameters a,b,c,d embody the prior knowledge and information about α and β. On this basis, the joint posterior distribution is

(12) $$\pi \left(\alpha ,\beta ,\vec{X}\right)\;\alpha^{D+a-1}\beta^{D+c-1}e^{\beta (E(\alpha )-d)-b\alpha }\prod_{j=1}^{D}x_{j}^{\alpha -1}{\left(1-x_{j}^{\alpha }\right)}^{\beta (1+R_{j})-1},$$

where $\vec{X}$ are observations $X_{1},X_{2},\ldots$. The conditional posterior distribution is defined by

$$\pi \left(\alpha ,\beta |\vec{X}\right)=\frac{\pi (\alpha ,\beta ,\vec{X})}{\int_{0}^{\infty }\int_{0}^{\infty }\pi \left(\alpha ,\beta ,\vec{X}\right)d\alpha d\beta }.$$

Under the SEL function, the Bayes estimators can be gained as

$${\hat{\psi }}_{S}=\frac{\int_{0}^{\infty }\int_{0}^{\infty }\psi \pi \left(\alpha ,\beta ,\vec{X}\right)d\alpha d\beta }{\int_{0}^{\infty }\int_{0}^{\infty }\pi \left(\alpha ,\beta ,\vec{X}\right)d\alpha d\beta },$$

where ψ stands for α,β or reliability function.

Under the LL function, the Bayes estimators are obtained as

$${\hat{\psi }}_{L}=-\frac{1}{p}\log\{\frac{\int_{0}^{\infty }\int_{0}^{\infty }e^{-p\psi }\pi \left(\alpha ,\beta ,\vec{X}\right)d\alpha d\beta }{\int_{0}^{\infty }\int_{0}^{\infty }\pi \left(\alpha ,\beta ,\vec{X}\right)d\alpha d\beta }\},$$

Under the EL function, the Bayes estimators are written as

$${\hat{\psi }}_{E}={\left[\frac{\int_{0}^{\infty }\int_{0}^{\infty }\psi^{-q}\pi \left(\alpha ,\beta ,\vec{X}\right)d\alpha d\beta }{\int_{0}^{\infty }\int_{0}^{\infty }\pi \left(\alpha ,\beta ,\vec{X}\right)d\alpha d\beta }\right]}^{-\frac{1}{q}}.$$

By observing the above Bayes estimation expressions, we find that they are all the ratios of two integrals and the explicit expressions are hard to acquire. Therefore, proper methods to approximate the above integrals need to be employed. Thus, we introduce Lindley’s approximation, as well as the Tierney and Kadane method, to get the closed estimators.

### 3.3. Lindley’s Approximation

Lindley [19] deduced this general term formula by developing asymptotic expansions for the ratio of integrals. To apply this method, we denote $\mu =(\mu_{1},\mu_{2})$ and consider the function $g(\mu_{1},\mu_{2})$ in the form of ratio of intergrals given by

(13) $$E\left(g\left(\mu_{1},\mu_{2}\right)\right)=\frac{\int_{0}^{\infty }\int_{0}^{\infty }g\left(\mu_{1},\mu_{2}\right)\pi \left(\mu_{1},\mu_{2},\vec{X}\right)d\mu_{1}d\mu_{2}}{\int_{0}^{\infty }\int_{0}^{\infty }\pi \left(\mu_{1},\mu_{2},\vec{X}\right)d\mu_{1}d\mu_{2}}=\frac{\int_{0}^{\infty }\int_{0}^{\infty }g\left(\mu_{1},\mu_{2}\right)e^{l\left(\mu_{1},\mu_{2}|\vec{X}\right)+\kappa \left(\mu_{1},\mu_{2}\right)}d\mu_{1}d\mu_{2}}{\int_{0}^{\infty }\int_{0}^{\infty }e^{l\left(\mu_{1},\mu_{2}|\vec{X}\right)+\kappa \left(\mu_{1},\mu_{2}\right)}d\mu_{1}d\mu_{2}},$$

where $g(\mu_{1},\mu_{2})$ is a certain function of $\mu_{1}$ and $\mu_{2}$, $l(\mu_{1},\mu_{2}|\vec{X})$ is the log-likelihood function and $\kappa \left(\mu_{1},\mu_{2}\right)=\log\pi \left(\mu_{1},\mu_{2}\right)$. Employing the Lindley method, the function $g(\mu_{1},\mu_{2})$ can be written as

(14) $$\hat{g}=g\left({\hat{\mu }}_{1},{\hat{\mu }}_{2}\right)+0.5\left[A+l_{03}B_{21}+l_{30}B_{12}+l_{12}C_{21}+l_{21}C_{12}\right]+\kappa_{1}A_{12}+\kappa_{2}A_{21},$$

where

$$\begin{array}{cc} \; & A=\sum_{i=1}^{2}\sum_{j=1}^{2}w_{ij}\tau_{ij},\;l_{ij}=\frac{\partial^{i+j}l\left(\mu_{1},\mu_{2}\right)}{\partial \mu_{1}^{i}\partial \mu_{2}^{j}},\;i,j=0,1,2,3,\;i+j=3,\\ \; & \kappa_{i}=\frac{\partial \kappa }{\partial \mu_{i}},\;w_{i}=\frac{\partial g}{\partial \mu_{i}},\;w_{ij}=\frac{\partial^{2}g}{\partial \mu_{i}\mu_{j}},\;A_{ij}=w_{i}\tau_{ii}+w_{j}\tau_{ji},\\ \; & B_{ij}=\left(w_{i}\tau_{ii}+w_{j}\tau_{ij}\right)\tau_{ii},\;C_{ij}=3w_{i}\tau_{ii}\tau_{ij}+w_{j}\left(\tau_{ii}\tau_{jj}+2\tau_{ij}^{2}\right). \end{array}$$

We further observe that $\tau_{ij}=\left(i,j\right)$th element of the inverse matrix ${\left[-\frac{\partial^{2}l\left(\mu_{1},\mu_{2}|\vec{X}\right)}{\partial \mu_{1}^{i}\partial \mu_{2}^{j}}\right]}^{-1}$.

For our estimation problem, μ=(α,β) and now let us deduce it in detail. For convenience, we denote

$$\tau_{11}=\frac{H}{M},\;\tau_{22}=\frac{G}{M},\;\tau_{12}=\tau_{21}=-\frac{I}{M},\;M=GH-I^{2},$$

where

$$\begin{array}{cc} G & =-\frac{\partial^{2}l}{\partial \alpha^{2}}=\frac{D}{\alpha^{2}}-\beta \left[\sum_{j=1}^{D}\left(1+R_{j}\right)\frac{-x_{j}^{\alpha }{\left(\log x_{j}\right)}^{2}}{{\left(1-x_{j}^{\alpha }\right)}^{2}}+E^{\left(2\right)}\left(\alpha \right)\right]-\sum_{j=1}^{D}\frac{x_{j}^{\alpha }{\left(\log x_{j}\right)}^{2}}{{\left(1-x_{j}^{\alpha }\right)}^{2}},\\ H & =-\frac{\partial^{2}l}{\partial \beta^{2}}=\frac{D}{\beta^{2}},\;I=-\frac{\partial^{2}l}{\partial \alpha \partial \beta }=-\left[\sum_{j=1}^{D}\left(1+R_{j}\right)\frac{-x_{j}^{\alpha }\log x_{j}}{1-x_{j}^{\alpha }}+E^{\left(1\right)}\left(\alpha \right)\right]. \end{array}$$

Also, for our problem we have

$$\begin{array}{cc} l_{30}=\frac{\partial^{3}l}{\partial \alpha^{3}}= & \frac{2D}{\alpha^{3}}+\beta \left[\sum_{j=1}^{D}\left(1+R_{j}\right){\left(\log x_{j}\right)}^{3}\frac{-x_{j}^{\alpha }\left(1+x_{j}^{\alpha }\right)}{{\left(1-x_{j}^{\alpha }\right)}^{3}}+E^{\left(3\right)}\left(\alpha \right)\right]\\ \; & +\sum_{j=1}^{D}{\left(\log x_{j}\right)}^{3}\frac{x_{j}^{\alpha }\left(1+x_{j}^{\alpha }\right)}{{\left(1-x_{j}^{\alpha }\right)}^{3}},\\ l_{21}= & \frac{\partial^{3}l}{\partial \alpha^{2}\partial \beta }=\sum_{j=1}^{D}\left(1+R_{j}\right)\frac{-x_{j}^{\alpha }{\left(\log x_{j}\right)}^{2}}{{\left(1-x_{j}^{\alpha }\right)}^{2}}+E^{\left(2\right)}\left(\alpha \right),\\ l_{12}= & \frac{\partial^{3}l}{\partial \alpha \partial \beta^{2}}=0,\;l_{03}=\frac{\partial^{3}l}{\partial \beta^{3}}=\frac{2D}{\beta^{3}},\\ \kappa_{1}= & \frac{a-1}{\alpha }-b,\;\kappa_{2}=\frac{c-1}{\beta }-d, \end{array}$$

where

$$E^{\left(2\right)}\left(\alpha \right)=\left\{\begin{array}{cc} 0 & \mathrm{for}\;\mathrm{Case}\;I\;\mathrm{and}\;\mathrm{III},\\ -R_{J+1}^{*}\frac{T^{\alpha }{\left(\log T\right)}^{2}}{{\left(1-T^{\alpha }\right)}^{2}} & \mathrm{for}\;\mathrm{Case}\;\mathrm{II}, \end{array}\right.$$

$$E^{\left(3\right)}\left(\alpha \right)=\left\{\begin{array}{cc} 0 & \mathrm{for}\;\mathrm{Case}\;I\;\mathrm{and}\;\mathrm{III},\\ -R_{J+1}^{*}\frac{T^{\alpha }{\left(\log T\right)}^{3}\left(1+T^{\alpha }\right)}{{\left(1-T^{\alpha }\right)}^{3}} & \mathrm{for}\;\mathrm{Case}\;\mathrm{II}. \end{array}\right.$$

- **Under the SEL function**,
  - when g(α,β)=α, we observe that
    $$w_{1}=1,\;w_{2}=w_{11}=w_{12}=w_{21}=w_{22}=0.$$
    Using the above Equation (14), the Bayes estimator of α can be obtained as
    $${\hat{\alpha }}_{S}=\hat{\alpha }+\frac{1}{M}\left(H\kappa_{1}-I\kappa_{2}\right)+\frac{0.5}{M^{2}}\left[H^{2}l_{30}-GIl_{03}-3HIl_{21}\right].$$
  - When g(α,β)=β, we can derive that
    $$w_{2}=1,\;w_{1}=w_{11}=w_{12}=w_{21}=w_{22}=0.$$
    Likewise, the Bayes estimator of β can be gained as
    $${\hat{\beta }}_{S}=\hat{\beta }+\frac{1}{M}\left(-I\kappa_{1}+G\kappa_{2}\right)+\frac{0.5}{M^{2}}\left[-HIl_{30}+G^{2}l_{03}+\left(HG+2I^{2}\right)l_{21}\right].$$
  - Let g(α,β)=R(t), we have
    $$\begin{array}{cc} w_{1} & =\beta {\left(1-t^{\alpha }\right)}^{\beta -1}\left(-t^{\alpha }\log t\right),\;w_{2}={\left(1-t^{\alpha }\right)}^{\beta }\log\left(1-t^{\alpha }\right),\\ w_{11} & =\beta t^{\alpha }{\left(\log t\right)}^{2}{\left(1-t^{\alpha }\right)}^{\beta -2}\left(\beta t^{\alpha }-1\right),\;w_{22}={\left(1-t^{\alpha }\right)}^{\beta }{\left[\log(1-t^{\alpha })\right]}^{2},\\ w_{12} & =w_{21}=-t^{\alpha }\log t{\left(1-t^{\alpha }\right)}^{\beta -1}\left[1+\beta \log(1-t^{\alpha })\right]. \end{array}$$
    The Bayes estimator of R(t) can be computed by
    $$\begin{array}{cc} {\hat{R}}_{S}=\hat{R}\left(t\right) & +\frac{0.5}{M^{2}}[M\left(Hw_{11}-2Iw_{12}+Gw_{22}\right)+H\left(Hw_{1}-Iw_{2}\right)l_{30}\\ \; & +G\left(Gw_{2}-Iw_{1}\right)l_{03}+\left(-3HIw_{1}+HGw_{2}+2I^{2}w_{2}\right)l_{21}]\\ \; & +\frac{1}{M}\left[\left(Hw_{1}-Iw_{2}\right)\kappa_{1}+\left(Gw_{2}-Iw_{1}\right)\kappa_{2}\right]. \end{array}$$
- **Under the LL function**,
  - when $g\left(\alpha ,\beta \right)=e^{-p\alpha }$, we observe that
    $$w_{1}=-pe^{-p\alpha },\;w_{11}=p^{2}e^{-p\alpha },\;w_{2}=w_{22}=w_{12}=w_{21}=0.$$
    The Bayes estimator of α can be obtained as
    $$\begin{array}{cc} {\hat{\alpha }}_{L}= & -\frac{1}{p}\log\{e^{-p\hat{\alpha }}+\frac{0.5}{M^{2}}\left[MHw_{11}+H^{2}w_{1}l_{30}-GIw_{1}l_{03}-3HIw_{1}l_{21}\right]\\ \; & +\frac{1}{M}\left(Hw_{1}\kappa_{1}-Iw_{1}\kappa_{2}\right)\}. \end{array}$$
  - When $g\left(\alpha ,\beta \right)=e^{-p\beta }$, we can derive that
    $$w_{2}=-pe^{-p\beta },\;w_{22}=p^{2}e^{-p\beta },\;w_{1}=w_{11}=w_{12}=w_{21}=0.$$
    Similarly, the Bayes estimator of β is in the form of
    $$\begin{array}{cc} {\hat{\beta }}_{L}= & -\frac{1}{p}\log\{e^{-p\hat{\beta }}+\frac{0.5}{M^{2}}\left[MGw_{22}-HIw_{2}l_{30}+G^{2}w_{2}l_{03}+\left(HG+2I^{2}\right)w_{2}l_{21}\right]\\ \; & +\frac{1}{M}\left(-Iw_{2}\kappa_{1}+Gw_{2}\kappa_{2}\right)\}. \end{array}$$
  - Let $g\left(\alpha ,\beta \right)=e^{-p{\left(1-t^{\alpha }\right)}^{\beta }}$, we have
    $$\begin{array}{cc} w_{1}= & p\beta \left(\log t\right)t^{\alpha }{\left(1-t^{\alpha }\right)}^{\beta -1}e^{-p{\left(1-t^{\alpha }\right)}^{\beta }},\\ w_{2}= & -p{\left(1-t^{\alpha }\right)}^{\beta }e^{-p{\left(1-t^{\alpha }\right)}^{\beta }}\log\left(1-t^{\alpha }\right),\\ w_{11}= & p\beta t^{\alpha }{\left(\log t\right)}^{2}{\left(1-t^{\alpha }\right)}^{\beta -2}e^{-p{\left(1-t^{\alpha }\right)}^{\beta }}\left[1-\beta t^{\alpha }+p\beta t^{\alpha }{\left(1-t^{\alpha }\right)}^{\beta }\right],\\ w_{22}= & -p{\left[\log(1-t^{\alpha })\right]}^{2}{\left(1-t^{\alpha }\right)}^{\beta }e^{-p{\left(1-t^{\alpha }\right)}^{\beta }}\left[1-p{\left(1-t^{\alpha }\right)}^{\beta }\right],\\ w_{12}= & w_{21}\\ = & p\left(\log t\right)t^{\alpha }{\left(1-t^{\alpha }\right)}^{\beta -1}e^{-p{\left(1-t^{\alpha }\right)}^{\beta }}\left[1+\beta \log\left(1-t^{\alpha }\right)-p\beta {\left(1-t^{\alpha }\right)}^{\beta }\log\left(1-t^{\alpha }\right)\right]. \end{array}$$
    The Bayes estimator of R(t) are acquired as
    $$\begin{array}{cc} {\hat{R}}_{L}= & -\frac{1}{p}\log\{e^{-p\hat{R}\left(t\right)}+\frac{0.5}{M^{2}}[M\left(Hw_{11}-2Iw_{12}+Gw_{22}\right)+H\left(Hw_{1}-Iw_{2}\right)l_{30}\\ \; & +G\left(Gw_{2}-Iw_{1}\right)l_{03}+\left(-3HIw_{1}+\left(HG+2I^{2}\right)w_{2}\right)l_{21}]\\ \; & +\frac{1}{M}\left[\left(Hw_{1}-Iw_{2}\right)\kappa_{1}+\left(Gw_{2}-Iw_{1}\right)\kappa_{2}\right]\}. \end{array}$$
- **Under the EL function**,
  - when $g\left(\alpha ,\beta \right)=\alpha^{-q}$, we observe that
    $$\begin{array}{c} w_{1}=-q\alpha^{-q-1},\;w_{11}=q\left(q+1\right)\alpha^{-q-2},\;w_{2}=w_{22}=w_{12}=w_{21}=0. \end{array}$$
    The Bayes estimator of α can be obtained as
    $${\hat{\alpha }}_{E}={\left\{{\hat{\alpha }}^{-q}+\frac{0.5}{M^{2}}\left[MHw_{11}+H^{2}w_{1}l_{30}-GIw_{1}l_{03}-3HIw_{1}l_{21}\right]+\frac{1}{M}\left(Hw_{1}\kappa_{1}-Uw_{1}\kappa_{2}\right)\right\}}^{-\frac{1}{q}}.$$
  - When $g\left(\alpha ,\beta \right)=\beta^{-q}$, we can derive that
    $$w_{2}=-q\beta^{-q-1},\;w_{22}=q\left(q+1\right)\beta^{-q-2},\;w_{1}=w_{11}=w_{12}=w_{21}=0.$$
    Analogously, the Bayes estimator of β is in the form of
    $$\begin{array}{cc} {\hat{\beta }}_{E}= & \{{\hat{\beta }}^{-q}+\frac{0.5}{M^{2}}\left[MGw_{2}-HIw_{2}l_{30}+G^{2}w_{2}l_{03}+\left(HG+2I^{2}\right)w_{2}l_{21}\right]\\ \; & +\frac{1}{M}\left(-Iw_{2}\kappa_{1}+Gw_{2}\kappa_{2}\right){\}}^{-\frac{1}{q}}. \end{array}$$
  - Let $g\left(\alpha ,\beta \right)={\left(1-t^{\alpha }\right)}^{-q\beta }$, we have
    $$\begin{array}{cc} \; & w_{1}=q\beta t^{\alpha }{\left(1-t^{\alpha }\right)}^{-q\beta -1}\log t,\;w_{2}=-q{\left(1-t^{\alpha }\right)}^{-q\beta }\log\left(1-t^{\alpha }\right),\\ \; & w_{11}=q\beta t^{\alpha }{\left(\log t\right)}^{2}{\left(1-t^{\alpha }\right)}^{-q\beta -2}\left(1+q\beta t^{\alpha }\right),\;w_{22}=q^{2}{\left[\log(1-t^{\alpha })\right]}^{2}{\left(1-t^{\alpha }\right)}^{-q\beta },\\ \; & w_{12}=w_{21}=qt^{\alpha }\log t{\left(1-t^{\alpha }\right)}^{-q\beta -1}\left[1-q\beta \log(1-t^{\alpha })\right]. \end{array}$$
    The Bayes estimate of R(t) can be achieved by
    $$\begin{array}{cc} {\hat{R}}_{L}= & \{\hat{R}{\left(t\right)}^{-q}+\frac{0.5}{M^{2}}[M\left(Hw_{11}-2Iw_{12}+Gw_{22}\right)+H\left(Hw_{1}-Iw_{2}\right)l_{30}\\ \; & +G\left(Gw_{2}-Iw_{1}\right)l_{03}+\left(-3HIw_{1}+\left(HG+2I^{2}\right)w_{2}\right)l_{21}]\\ \; & +\frac{1}{M}\left[\left(Hw_{1}-Iw_{2}\right)\kappa_{1}+\left(Gw_{2}-Iw_{1}\right)\kappa_{2}\right]{\}}^{-\frac{1}{q}}. \end{array}$$

### 3.4. Tierney and Kadane Method

Besides Lindley’s approximation, Tierney and Kadane [20] proposed another approach to approximate integrals and Howlader and Hossain [21] made a comparison between these two methods of Pareto distribution under several diverse censored sample. Recall that the expectation of posterior of g(α,β) is

(15) $$\hat{g}=E\left(g\left(\alpha ,\beta \right)\right)=\frac{\int_{0}^{\infty }\int_{0}^{\infty }g\left(\alpha ,\beta \right)e^{l\left(\alpha ,\beta |\vec{X}\right)+\kappa \left(\alpha ,\beta \right)}d\alpha d\beta }{\int_{0}^{\infty }\int_{0}^{\infty }e^{l\left(\alpha ,\beta |\vec{X}\right)+\kappa \left(\alpha ,\beta \right)}d\alpha d\beta }=\frac{\int_{0}^{\infty }\int_{0}^{\infty }e^{n\delta_{g}^{*}\left(\alpha ,\beta \right)}d\alpha d\beta }{\int_{0}^{\infty }\int_{0}^{\infty }e^{n\delta (\alpha ,\beta )}d\alpha d\beta },$$

where

$$\delta \left(\alpha ,\beta \right)=\frac{l\left(\alpha ,\beta |\vec{X}\right)+\kappa \left(\alpha ,\beta \right)}{n},\;\delta_{g}^{*}\left(\alpha ,\beta \right)=\delta \left(\alpha ,\beta \right)+\frac{\log g(\alpha ,\beta )}{n},$$

with κ(α,β)=logπ(α,β) and $l(\alpha ,\beta |\vec{X})$ denoting the likelihood funciton of α and β. Suppose that $\left({\hat{\alpha }}_{\delta },{\hat{\beta }}_{\delta }\right)$ represents the values of (α,β) which maximize δ(α,β) and $\left({\hat{\alpha }}_{\delta^{*}},{\hat{\beta }}_{\delta^{*}}\right)$ represent the values of (α,β) which maximize $\delta_{g}^{*}\left(\alpha ,\beta \right)$. Thus, the approximation of the above Equation (15) is

(16) $$\hat{g}=\sqrt{\frac{|\Omega_{g}^{*}|}{\left|\Omega \right|}}e^{n\left[\delta_{g}^{*}\left({\hat{\alpha }}_{\delta^{*}},{\hat{\beta }}_{\delta^{*}}\right)-\delta \left({\hat{\alpha }}_{\delta },{\hat{\beta }}_{\delta }\right)\right]}.$$

Here, |Ω| and $|\Omega_{g}^{*}|$ are the negatives of inverse Hessians of δ and $\delta_{g}^{*}$ respectively calculated at $\left({\hat{\alpha }}_{\delta },{\hat{\beta }}_{\delta }\right)$ as well as $\left({\hat{\alpha }}_{\delta^{*}},{\hat{\beta }}_{\delta^{*}}\right)$. It is worth noting that |Ω| and $\delta ({\hat{\alpha }}_{\delta },{\hat{\beta }}_{\delta })$ in Equation (16) do not rely on *g*, whereas $|\Omega_{g}^{*}|$ and $\delta_{g}^{*}\left({\hat{\alpha }}_{\delta^{*}},{\hat{\beta }}_{\delta^{*}}\right)$ do rely on *g*. Below the Bayes estimators of parameter α, β and R(t) are acquired employing this method.

For the two-parameter Kumaraswamy distribution, we have

$$\begin{array}{cc} \delta \left(\alpha ,\beta \right)=\frac{1}{n} & \{D\log\alpha \beta +\left(a-1\right)\log\alpha +\alpha \left(\sum_{j=1}^{D}\log x_{j}-b\right)+\left(c-1\right)\log\beta \\ \; & +\beta \left[\sum_{j=1}^{D}\left(1+R_{j}\right)\log\left(1-x_{j}^{\alpha }\right)+E\left(\alpha \right)-d\right]-\sum_{j=1}^{D}\log\left(1-x_{j}^{\alpha }\right)-\sum_{j=1}^{D}\log x_{j}\}. \end{array}$$

Subsequently, the following set of equations need to be solved to get $\left({\hat{\alpha }}_{\delta },{\hat{\beta }}_{\delta }\right)$:

$$\begin{array}{cc} \; & \frac{\partial \delta }{\partial \alpha }=\frac{1}{n}\left\{\frac{D+a-1}{\alpha }+\beta \left[\sum_{j=1}^{D}\left(1+R_{j}\right)\frac{-x_{j}^{\alpha }\log x_{j}}{1-x_{j}^{\alpha }}+E^{\left(1\right)}\left(\alpha \right)\right]+\sum_{j=1}^{D}\frac{\log x_{j}}{1-x_{j}^{\alpha }}-b\right\}=0,\\ \; & \frac{\partial \delta }{\partial \beta }=\frac{1}{n}\left\{\frac{D+c-1}{\beta }+\sum_{j=1}^{D}\left(1+R_{j}\right)\log\left(1-x_{j}^{\alpha }\right)+E\left(\alpha \right)-d\right\}=0. \end{array}$$

Then, we obtain |Ω|, which is given by

$$\left|\Omega \right|={\left[\frac{\partial^{2}\delta }{\partial \alpha^{2}}\times \frac{\partial^{2}\delta }{\partial \beta^{2}}-\frac{\partial^{2}\delta }{\partial \alpha \partial \beta }\times \frac{\partial^{2}\delta }{\partial \beta \partial \alpha }\right]}^{-1},$$

where

$$\begin{array}{cc} \frac{\partial^{2}\delta }{\partial \alpha^{2}} & =\frac{1}{n}\{-\frac{D+\alpha -1}{\alpha^{2}}+\beta \left[\sum_{j=1}^{D}\left(1+R_{j}\right){\left(\log x_{j}\right)}^{2}\frac{-x_{j}^{\alpha }}{{\left(1-x_{j}^{\alpha }\right)}^{2}}+E^{\left(2\right)}\left(\alpha \right)\right]\\ \; & +\sum_{j=1}^{D}\frac{x_{j}^{\alpha }{\left(\log x_{j}\right)}^{2}}{{\left(1-x_{j}^{\alpha }\right)}^{2}}\},\\ \frac{\partial^{2}\delta }{\partial \beta^{2}} & =-\frac{D+c-1}{n\beta^{2}},\;\frac{\partial^{2}\delta }{\partial \alpha \partial \beta }=\frac{1}{n}\left[\sum_{j=1}^{D}\left(1+R_{j}\right)\frac{-x_{j}^{\alpha }\log x_{j}}{1-x_{j}^{\alpha }}+E^{\left(1\right)}\left(\alpha \right)\right]. \end{array}$$

Recall that expressions $|\Omega_{g}^{*}|$ and $\delta_{g}^{*}$ in Equation (16) rely on function *g*.

- **Under the SEL function**,
  - g(α,β)=α for α and corresponding function $\delta^{*}$ comes to be
    $$\delta_{\alpha }^{*}=\delta +\frac{\log\alpha }{n}.$$
    Later, we solve the set of following equations
    $$\frac{\partial \delta_{\alpha }^{*}}{\partial \alpha }=\frac{\partial \delta }{\partial \alpha }+\frac{1}{n\alpha }=0,\;\frac{\partial \delta_{\alpha }^{*}}{\partial \beta }=\frac{\partial \delta }{\partial \beta }=0,$$
    and gain $\left({\hat{\alpha }}_{\delta^{*}},{\hat{\beta }}_{\delta^{*}}\right)$. Then, $|\Omega_{\alpha }^{*}|$ is computed as
    $$|\Omega_{\alpha }^{*}|={\left[\frac{\partial^{2}\delta_{\alpha }^{*}}{\partial \alpha^{2}}\times \frac{\partial^{2}\delta_{\alpha }^{*}}{\partial \beta^{2}}-\frac{\partial^{2}\delta_{\alpha }^{*}}{\partial \alpha \partial \beta }\times \frac{\partial^{2}\delta_{\alpha }^{*}}{\partial \beta \partial \alpha }\right]}^{-1},$$
    where
    $$\frac{\partial^{2}\delta_{\alpha }^{*}}{\partial \alpha^{2}}=\frac{\partial^{2}\delta }{\partial \alpha^{2}}-\frac{1}{n\alpha^{2}},\;\frac{\partial^{2}\delta_{\alpha }^{*}}{\partial \beta^{2}}=\frac{\partial^{2}\delta }{\partial \beta^{2}},\;\frac{\partial^{2}\delta_{\alpha }^{*}}{\partial \alpha \partial \beta }=\frac{\partial^{2}\delta_{\alpha }^{*}}{\partial \beta \partial \alpha }=\frac{\partial^{2}\delta }{\partial \alpha \partial \beta }.$$
    Using the above expression in Equation (16), the desired Bayes estimator of α can be written as
    $${\hat{\alpha }}_{S}=\sqrt{\frac{|\Omega_{\alpha }^{*}|}{\left|\Omega \right|}}e^{n\left[\delta_{\alpha }^{*}\left({\hat{\alpha }}_{\delta^{*}},{\hat{\beta }}_{\delta^{*}}\right)-\delta \left({\hat{\alpha }}_{\delta },{\hat{\beta }}_{\delta }\right)\right]}.$$
    The Bayes estimator of β based on the SEL function can be attained in similiar way.
  - Now, we consider the relibility function R(t). Let $g\left(\alpha ,\beta \right)={\left(1-t^{\alpha }\right)}^{\beta }$, then we have
    $$\delta_{Rt}^{*}=\delta +\frac{\beta \log(1-t^{\alpha })}{n}.$$
    Hence, we calculate $\left({\hat{\alpha }}_{\delta^{*}},{\hat{\beta }}_{\delta^{*}}\right)$ by figuring out the following set of equations:
    $$\frac{\partial \delta_{Rt}^{*}}{\partial \alpha }=\frac{\partial \delta }{\partial \alpha }-\frac{\beta t^{\alpha }\log t}{n(1-t^{\alpha })}=0,\;\frac{\partial \delta_{Rt}^{*}}{\partial \beta }=\frac{\partial \delta }{\partial \beta }+\frac{\log(1-t^{\alpha })}{n}=0.$$
    Subsequently, we deduce $|\Omega_{Rt}^{*}|$ as follows:
    $$|\Omega_{Rt}^{*}|={\left[\frac{\partial^{2}\delta_{Rt}^{*}}{\partial \alpha^{2}}\times \frac{\partial^{2}\delta_{Rt}^{*}}{\partial \beta^{2}}-\frac{\partial^{2}\delta_{Rt}^{*}}{\partial \alpha \partial \beta }\times \frac{\partial^{2}\delta_{Rt}^{*}}{\partial \beta \partial \alpha }\right]}^{-1},$$
    where
    $$\begin{array}{cc} \; & \frac{\partial^{2}\delta_{Rt}^{*}}{\partial \alpha^{2}}=\frac{\partial^{2}\delta }{\partial \alpha^{2}}-\frac{\beta t^{\alpha }{\left(\log t\right)}^{2}}{n{\left(1-t^{\alpha }\right)}^{2}},\;\frac{\partial^{2}\delta_{Rt}^{*}}{\partial \beta^{2}}=\frac{\partial^{2}\delta }{\partial \beta^{2}},\;\\ \; & \frac{\partial^{2}\delta_{Rt}^{*}}{\partial \alpha \partial \beta }=\frac{\partial^{2}\delta_{Rt}^{*}}{\partial \beta \partial \alpha }=\frac{\partial^{2}\delta }{\partial \alpha \partial \beta }-\frac{t^{\alpha }\log t}{n(1-t^{\alpha })}. \end{array}$$
    After that, the Bayes estimator of reliability function turns out to be
    $${\hat{R}}_{S}=\sqrt{\frac{|\Omega_{Rt}^{*}|}{\left|\Omega \right|}}e^{n\left[\delta_{Rt}^{*}\left({\hat{\alpha }}_{\delta^{*}},{\hat{\beta }}_{\delta^{*}}\right)-\delta \left({\hat{\alpha }}_{\delta },{\hat{\beta }}_{\delta }\right)\right]}.$$
- **Under the LL function**,
  - $g\left(\alpha ,\beta \right)=e^{-p\alpha }$ for α and corresponding function $\delta^{*}$ turns into
    $$\delta_{\alpha }^{*}\left(\alpha ,\beta \right)=\delta \left(\alpha ,\beta \right)-\frac{p\alpha }{n}.$$
    Later, we solve the set of following equations
    $$\frac{\partial \delta_{\alpha }^{*}}{\partial \alpha }=\frac{\partial \delta }{\partial \alpha }-\frac{p}{n}=0,\;\frac{\partial \delta_{\alpha }^{*}}{\partial \beta }=\frac{\partial \delta }{\partial \beta }=0,$$
    and gain $\left({\hat{\alpha }}_{\delta^{*}},{\hat{\beta }}_{\delta^{*}}\right)$. Then, $|\Omega_{\alpha }^{*}|$ is computed as
    $$|\Omega_{\alpha }^{*}|={\left[\frac{\partial^{2}\delta_{\alpha }^{*}}{\partial \alpha^{2}}\times \frac{\partial^{2}\delta_{\alpha }^{*}}{\partial \beta^{2}}-\frac{\partial^{2}\delta_{\alpha }^{*}}{\partial \alpha \partial \beta }\times \frac{\partial^{2}\delta_{\alpha }^{*}}{\partial \beta \partial \alpha }\right]}^{-1},$$
    where
    $$\frac{\partial^{2}\delta_{\alpha }^{*}}{\partial \alpha^{2}}=\frac{\partial^{2}\delta }{\partial \alpha^{2}},\;\frac{\partial^{2}\delta_{\alpha }^{*}}{\partial \beta^{2}}=\frac{\partial^{2}\delta }{\partial \beta^{2}},\;\frac{\partial^{2}\delta_{\alpha }^{*}}{\partial \beta \partial \alpha }=\frac{\partial^{2}\delta_{\alpha }^{*}}{\partial \alpha \partial \beta }=\frac{\partial^{2}\delta }{\partial \alpha \partial \beta }.$$
    The Bayes estimator of α can be written as
    $${\hat{\alpha }}_{L}=-\frac{1}{p}\log\left\{\sqrt{\frac{|\Omega_{\alpha }^{*}|}{\left|\Omega \right|}}e^{n\left[\delta_{\alpha }^{*}\left({\hat{\alpha }}_{\delta^{*}},{\hat{\beta }}_{\delta^{*}}\right)-\delta \left({\hat{\alpha }}_{\delta },{\hat{\beta }}_{\delta }\right)\right]}\right\}.$$
    Likewise, based on the linex loss function, the Bayes estimator of β will be realized.
  - Now, we consider the relibility function R(t). Let $g\left(\alpha ,\beta \right)=e^{-p{\left(1-t^{\alpha }\right)}^{\beta }}$, then we have
    $$\delta_{Rt}^{*}=\delta -\frac{p{\left(1-t^{\alpha }\right)}^{\beta }}{n}.$$
    Hence, we calculated $\left({\hat{\alpha }}_{\delta^{*}},{\hat{\beta }}_{\delta^{*}}\right)$ by figuring out the following set of equations
    $$\frac{\partial \delta_{Rt}^{*}}{\partial \alpha }=\frac{\partial \delta }{\partial \alpha }+\frac{p\beta t^{\alpha }{\left(1-t^{\alpha }\right)}^{\beta -1}\log t}{n}=0,\;\frac{\partial \delta_{Rt}^{*}}{\partial \beta }=\frac{\partial \delta }{\partial \beta }-\frac{p{\left(1-t^{\alpha }\right)}^{\beta }\log\left(1-t^{\alpha }\right)}{n}=0.$$
    Subsequently, we deduce $|\Omega_{Rt}^{*}|$ as follows:
    $$|\Omega_{Rt}^{*}|={\left[\frac{\partial^{2}\delta_{Rt}^{*}}{\partial \alpha^{2}}\times \frac{\partial^{2}\delta_{Rt}^{*}}{\partial \beta^{2}}-\frac{\partial^{2}\delta_{Rt}^{*}}{\partial \alpha \partial \beta }\times \frac{\partial^{2}\delta_{Rt}^{*}}{\partial \beta \partial \alpha }\right]}^{-1},$$
    where
    $$\begin{array}{cc} \; & \frac{\partial^{2}\delta_{Rt}^{*}}{\partial \alpha^{2}}=\frac{\partial^{2}\delta }{\partial \alpha^{2}}+\frac{p\beta t^{\alpha }{\left(\log t\right)}^{2}{\left(1-t^{\alpha }\right)}^{\beta -2}\left(1-\beta t^{\alpha }\right)}{n},\\ \; & \frac{\partial^{2}\delta_{Rt}^{*}}{\partial \beta^{2}}=\frac{\partial^{2}\delta }{\partial \beta^{2}}-\frac{p{\left[\log(1-t^{\alpha })\right]}^{2}{\left(1-t^{\alpha }\right)}^{\beta }}{n},\\ \; & \frac{\partial^{2}\delta_{Rt}^{*}}{\partial \alpha \partial \beta }=\frac{\partial^{2}\delta_{Rt}^{*}}{\partial \beta \partial \alpha }=\frac{\partial^{2}\delta }{\partial \alpha \partial \beta }+\frac{pt^{\alpha }\log t{\left(1-t^{\alpha }\right)}^{\beta -1}\left[1+\beta \log(1-t^{\alpha })\right]}{n}. \end{array}$$
    After that, the Bayes estimator of R(t) is
    $${\hat{R}}_{L}=-\frac{1}{p}\log\left\{\sqrt{\frac{|\Omega_{Rt}^{*}|}{\left|\Omega \right|}}e^{n\left[\delta_{Rt}^{*}\left({\hat{\alpha }}_{\delta^{*}},{\hat{\beta }}_{\delta^{*}}\right)-\delta \left({\hat{\alpha }}_{\delta },{\hat{\beta }}_{\delta }\right)\right]}\right\}.$$
- **Under the EL function**,
  - $g\left(\alpha ,\beta \right)=\alpha^{-q}$ for α and corresponding function $\delta^{*}$ becomes
    $$\delta_{\alpha }^{*}=\delta -\frac{q\log\alpha }{n}.$$
    Later, we solve the set of following equations
    $$\frac{\partial \delta_{\alpha }^{*}}{\partial \alpha }=\frac{\partial \delta }{\partial \alpha }-\frac{q}{n\alpha }=0,\;\frac{\partial \delta_{\alpha }^{*}}{\partial \beta }=\frac{\partial \delta }{\partial \beta }=0,$$
    and gain $\left({\hat{\alpha }}_{\delta^{*}},{\hat{\beta }}_{\delta^{*}}\right)$. Then, $|\Omega_{\alpha }^{*}|$ is computed as
    $$|\Omega_{\alpha }^{*}|={\left[\frac{\partial^{2}\delta_{\alpha }^{*}}{\partial \alpha^{2}}\times \frac{\partial^{2}\delta_{\alpha }^{*}}{\partial \beta^{2}}-\frac{\partial^{2}\delta_{\alpha }^{*}}{\partial \alpha \partial \beta }\times \frac{\partial^{2}\delta_{\alpha }^{*}}{\partial \beta \partial \alpha }\right]}^{-1},$$
    where
    $$\frac{\partial^{2}\delta_{\alpha }^{*}}{\partial \alpha^{2}}=\frac{\partial^{2}\delta }{\partial \alpha^{2}}+\frac{q}{n\alpha^{2}},\;\frac{\partial^{2}\delta_{\alpha }^{*}}{\partial \beta^{2}}=\frac{\partial^{2}\delta }{\partial \beta^{2}},\;\frac{\partial^{2}\delta_{\alpha }^{*}}{\partial \beta \partial \alpha }=\frac{\partial^{2}\delta_{\alpha }^{*}}{\partial \alpha \partial \beta }=\frac{\partial^{2}\delta }{\partial \alpha \partial \beta }.$$
    The desired Bayes estimator of α can be derived as
    $${\hat{\alpha }}_{E}=\sqrt{\frac{|\Omega_{\alpha }^{*}|}{\left|\Omega \right|}}e^{-\frac{n}{q}\left[\delta_{\alpha }^{*}\left({\hat{\alpha }}_{\delta^{*}},{\hat{\beta }}_{\delta^{*}}\right)-\delta \left({\hat{\alpha }}_{\delta },{\hat{\beta }}_{\delta }\right)\right]}.$$
    Obviously, under the EL function, we can also obtain the Bayes estimator of β.
  - Now, we consider the relibility function R(t). Let $g\left(\alpha ,\beta \right)={\left(1-t^{\alpha }\right)}^{-q\beta }$, then we have
    $$\delta_{Rt}^{*}=\delta -\frac{q\beta \log(1-t^{\alpha })}{n}.$$
    Hence, we calculate $\left({\hat{\alpha }}_{\delta^{*}},{\hat{\beta }}_{\delta^{*}}\right)$ by figuring out the following set of equations:
    $$\frac{\partial \delta_{Rt}^{*}}{\partial \alpha }=\frac{\partial \delta }{\partial \alpha }+\frac{q\beta t^{\alpha }\log t}{n(1-t^{\alpha })}=0,\;\frac{\partial \delta_{Rt}^{*}}{\partial \beta }=\frac{\partial \delta }{\partial \beta }-\frac{q\log(1-t^{\alpha })}{n}=0.$$
    Subsequently, we deduce $|\Omega_{Rt}^{*}|$ as follows:
    $$|\Omega_{Rt}^{*}|={\left[\frac{\partial^{2}\delta_{Rt}^{*}}{\partial \alpha^{2}}\times \frac{\partial^{2}\delta_{Rt}^{*}}{\partial \beta^{2}}-\frac{\partial^{2}\delta_{Rt}^{*}}{\partial \alpha \partial \beta }\times \frac{\partial^{2}\delta_{Rt}^{*}}{\partial \beta \partial \alpha }\right]}^{-1},$$
    where
    $$\begin{array}{cc} \; & \frac{\partial^{2}\delta_{Rt}^{*}}{\partial \alpha^{2}}=\frac{\partial^{2}\delta }{\partial \alpha^{2}}+\frac{q\beta t^{\alpha }{\left(\log t\right)}^{2}}{n{\left(1-t^{\alpha }\right)}^{2}},\\ \; & \frac{\partial^{2}\delta_{Rt}^{*}}{\partial \beta^{2}}=\frac{\partial^{2}\delta }{\partial \beta^{2}},\\ \; & \frac{\partial^{2}\delta_{Rt}^{*}}{\partial \beta \partial \alpha }=\frac{\partial^{2}\delta_{Rt}^{*}}{\partial \alpha \partial \beta }=\frac{\partial^{2}\delta }{\partial \alpha \partial \beta }+\frac{qt^{\alpha }\log t}{n(1-t^{\alpha })}. \end{array}$$
    After that, the Bayes estimator of R(t) results to be
    $${\hat{R}}_{L}=\sqrt{\frac{|\Omega_{Rt}^{*}|}{\left|\Omega \right|}}e^{-\frac{n}{q}\left[\delta_{Rt}^{*}\left({\hat{\alpha }}_{\delta^{*}},{\hat{\beta }}_{\delta^{*}}\right)-\delta \left({\hat{\alpha }}_{\delta },{\hat{\beta }}_{\delta }\right)\right]}.$$

## 4. Simulation Study

Within the simulation experiment, generating the generalized progressive hybrid censoring sample is our first step. Before continuing further, we give the way to generate progressive Type-II censoring sample in accordance with Balakrishnan and Sandhu [22]. He presented a simple but effective simulational algorithm, which enables one to collect a series of progressive Type-II censored sample out of any continuous distribution. By adapting this method, we give the algorithm of generating the generalized progressive hybrid censoring sample as below.

**Step-1:** Generate *m* independent observations $W_{1},W_{2},\ldots ,W_{m}$ which each follow the standard uniform distribution. **Step-2:** Set $Z_{i}=W_{i}^{\frac{1}{i+R_{m}+\ldots +R_{m-i+1}}}$, for i=1,…,m. **Step-3:** Set $Y_{i}=1-Z_{m}\ldots Z_{m-i+1}$, for i=1,…,m. Then, $Y_{1},\ldots ,Y_{m}$ are the desired progressive Type-II censored sample which comes from the standard uniform distribution.
**Step-4:** At last we set $X_{i}=F^{-1}\left(Y_{i}\right)$, for i=1,…,m, where $F^{-1}\left(Y_{i}\right)$ stands for the inverse culmulative density function of any distribution considered. $X_{1},X_{2},\ldots ,X_{m}$ are the desired progressive Type-II censored sample out of the distribution F(). In this article, F() is the Kumaraswamy distribution. **Step-4.1:** When $T<X_{k}<X_{m}$, the generalized progressive hybrid censored sample are $(X_{1},X_{2}$, *…*, $X_{k})$ (i.e., Case I);
**Step-4.2:** When $X_{k}<T<X_{m}$, *J* can be determinde which makes $X_{J}<T<X_{J+1}$, and the generalized progressive hybrid censored sample is $(X_{1},X_{2}$, *…*, $X_{J})$ (i.e., Case II); **Step-4.3:** When $X_{k}<X_{m}<T$, the generalized progressive hybrid censored sample is $(X_{1},X_{2}$, *…*, $X_{m})$ (i.e., Case III).

Without loss of generality, it is found to use the Kumaraswamy distribution which takes the value of α = 3, β = 2 and *T* = 0.9. The process is replicated 1000 times in each case. The results have been obtained under diverse (*n*, *m*, *k*). The censoring schemes employed in this simulation are

$$\begin{array}{cc} \mathrm{Scheme}\;I:\; & R_{1}=n-m,\;R_{2}=\ldots =R_{m}=0,\\ \mathrm{Scheme}\;\mathrm{II}:\; & R_{1}=\ldots =R_{m-1}=0,\;R_{m}=n-m,\\ \mathrm{Scheme}\;\mathrm{III}:\; & R_{1}=\left(n-m\right)/2,\;R_{2}=\ldots =R_{m-1}=0,\;R_{m}=\left(n-m\right)/2. \end{array}$$

We compute both the MLE and Bayes estimates. Bayes estimates are respectively on the condition of the non-informative prior distribution, which means that four hyperparameters *a*, *b*, *c*, *d* adopt values of 0, and informative prior distribution, where *a* = 1.5, *c* = 1, and *b* = *d* = 0.5. Bayes estimates are obtained under loss function subject to SEL, LL and EL functions. As for linex loss function, relative estimates are gained with *p* = 0.2, 0.8. General entropy loss function is considered with *q* = 0.2, 0.8. Finally, we use mean squared errors (MSEs), as well as absolute biases (ABs), to evaluate the accuracy of the estimations. They are

$$\begin{array}{cc} MSEs & =\frac{1}{S}\sum_{i=1}^{S}{\left(\hat{\sigma_{i}}-\sigma \right)}^{2},\\ ABs & =\frac{1}{S}\sum_{i=1}^{S}\left|\hat{\sigma_{i}}-\sigma \right|. \end{array}$$

where σ is the true value, $\hat{\sigma }$ stands for the corresponding estimate, and *S* represents the simulation times. The simulation results, Table A1–Table A3, are shown in the Appendix A. In every two rows of Table A1–Table A3, the upper one is MSEs and the lower one is ABs. We put the corresponding MSEs and ABs of MLE in the fifth column. Besides, all other columns are composed of eight values. The first two values represent the MSEs and ABs in light of Lindley approximation with noninformative prior distribution. The second two values represent the MSEs and ABs using Lindley approximation with informative prior distribution. The third two values denote the MSEs and ABs using TK method with noninformative prior distribution. The fourth two values correspond to the MSEs and ABs using TK method with informative prior distribution.

In Table A1, it can be seen that with the sample size *n* increasing, MSEs decrease, since as *n* increases, more additional information is gathered. For a given sample size, MSEs also decline with the generalized progressive hybrid censored sample $R_{i}$ decreasing, namely *m* increasing. Further, with *n* and *m* kept constant, the MSEs decline as the accepted bare minimum of failures *k* increases. In general, as for MSEs, Bayes estimates are superior to the MLEs, and there is no significant difference among the three schemes. Particularly, respective Bayes estimates of α under SEL make a better performance compared to the corresponding LL and EL. Besides, the Bayes estimates with informative prior provide better performance compared with the respective MSEs with noninformative prior. Estimation under LL with *p* = 0.2 gives a better option than *p* = 0.8, while *q* = 0.2 tends to produce lower MSEs than *q* = 0.8 under EL. Overall, with proper prior information, the Bayes estimates based on the SEL function and TK method do better than other estimates for α.

In Table A2, it can be seen that with the sample size *n* increasing, MSEs decrease, since as *n* increases, more additional information is gathered. For a given sample size, MSEs also decline with the generalized progressive hybrid censored sample $R_{i}$ decreasing, namely *m* increasing. Further, with *n* and *m* kept constant, the MSEs decline as the accepted bare minimum of failures *k* increases. In general, as for MSEs, Bayes estimates are superior to the MLEs, and there is no significant difference among the three schemes. Particularly, respective Bayes estimates of β under LL perform better than the corresponding SEL, as well as EL. Besides, the Bayes estimates with informative prior provide better performance compared with the respective MSEs with noninformative prior. Estimation under LL with *p* = 0.2 gives a better option than *p* = 0.8, while *q* = 0.2 tends to produce lower MSEs than *q* = 0.8 under EL. As for β, the Bayes estimates with proper prior based on the lines loss function and Lindley method perform better than other estimates.

In Table A3, it can be seen that with the sample size *n* increasing, MSEs decrease, since as *n* increases, more additional information is gathered. For a given sample size, MSEs also decline with the generalized progressive hybrid censored sample $R_{i}$ decreasing, namely *m* increasing. Further, with *n* and *m* kept constant, the MSEs decline as the accepted bare minimum of failures *k* increases. The maximum likelihood estimates outperform noninformative Bayes estimates, and there is no significant difference among the three schemes. Particularly, respective Bayes estimates of R(t) under LL perform worse than the corresponding SEL, as well as EL. Besides, the Bayes estimates with informative prior provide better performance compared with the respective MSEs with noninformative prior. In the case of LL, the choice *p* = 0.8 is preferred, while in the case of EL, the option *q* = 0.2 produces better results.

## 5. Data Analysis

**Figure 4:**
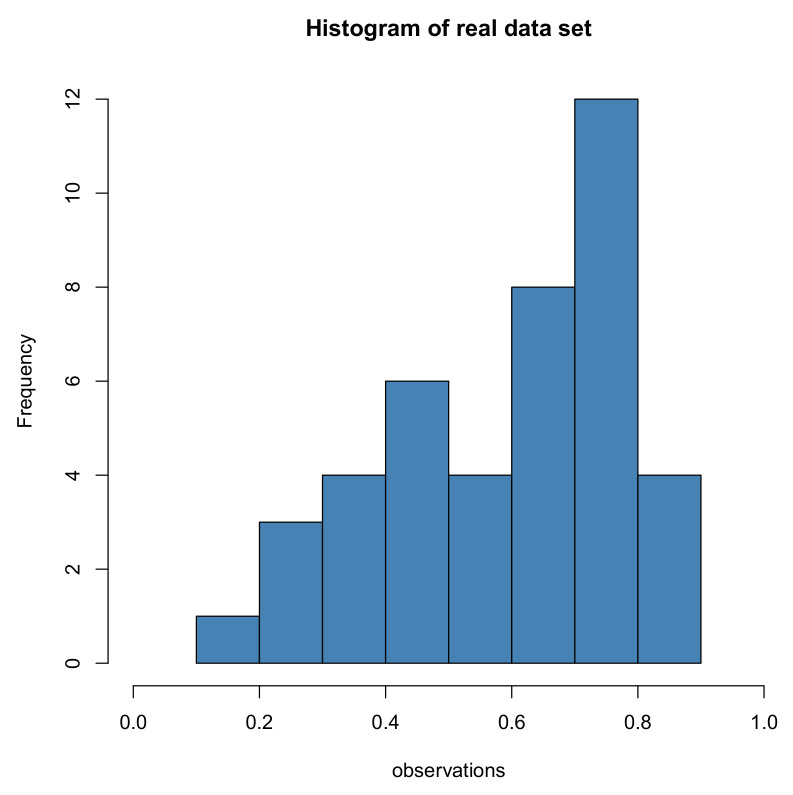
Histogram of real dataset.

This part illustrates a real-life dataset, aiming at analyzing the validity of the presented estimation methods. The following dataset shows the monthly water capacity of the Shasta reservoir in the time range of August and December from 1975 to 2016. These data have been used before by some statisticians, such as Kohansal [23]. Because K(α,β) is defined for 0<x<1, all data are divided by the total capacity of Shasta reservoir 4,552,000 acre-foot. The transformed data are tabulated in Table 1 and presented as a histogram in Figure 4.

One may doubt whether the considered dataset comes from the Kumaraswamy distribution or not. To verify the reasonableness, we fit the Kumaraswamy distribution to the dataset, competing with two other distributions—exponentiated exponential distribution (EED) and Lomax distribution (LD). The corresponding cdf and pdf are given below.

$$\begin{array}{cc} (EED)\;cdf:\; & F\left(x\right)={\left(1-e^{-\beta x}\right)}^{\alpha },\;\alpha ,\beta ,x>0,\\ pdf:\; & f\left(x\right)=\alpha \beta {\left(1-e^{-\beta x}\right)}^{\alpha -1}e^{-\beta x},\;\alpha ,\beta ,x>0,\\ (LD)\;cdf:\; & F\left(x\right)=1-{\left(1+\frac{x}{\beta }\right)}^{\alpha },\;\alpha ,\beta ,x>0,\\ pdf:\; & f\left(x\right)=\frac{\alpha }{\beta }{\left(1+\frac{x}{\beta }\right)}^{\alpha +1},\;\alpha ,\beta ,x>0. \end{array}$$

**Figure 5:**
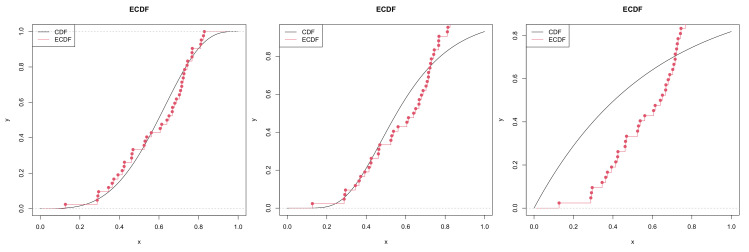
Empirical Cumulative Distributions. **Left** panel: K(α,β); **middle** panel: EED; **right** panel: LD.

**Figure 6:**
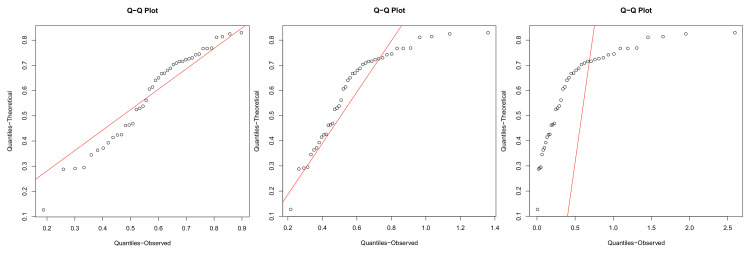
Quantile-Quantile Plots. **Left** panel: K(α,β); **middle** panel: EED; **right** panel: LD.

We employ the Akaike’s information criterion (AIC), defined by −2ln(L)+2k, the associated second order criterion (AICc), defined by $AIC+\frac{2k(k+1)}{n-k-1}$ and Bayesian information criterion (BIC), defined by −2ln(L)+kln(n), where *L* is maximized value of the likelihood function, *k* is the number of the parameters, *n* is the number of observations, as well as Kolmogorov–Smirnov (K–S) statistics with its *p*-value. The results are shown in Table 2. Besides, the empirical cumulative distribution functions and the quantile–quantile plots are given respectively in Figure 5 and Figure 6.

Considering that the Kumaraswamy distribution has lower AIC, AICc, BIC and K–S statistics and higher *p*-value, it is reasonable to say we fail to reject the hypothesis that the data come from Kumaraswamy.

The following data is generalized progressive hybrid censored sample artificially by using *m* = 21 and $R_{1}$ = $R_{2}$ = *…* = $R_{21}$ = 1. The ordered progressive Type-II censored dataset is given in Table 3.

In this illustration, take T = 0.75 and k = 19 for Case I, T = 0.75 and k = 14 for Case II, as well as T = 0.9 and k = 14 for Case III. Table 4 presents the estimation of α, β, as well as the reliability function of the generalized progressive hybrid censored sample.

## 6. Conclusions

In this paper, we estimate two parameters and the reliability of the Kumaraswamy distribution when the dataset is sampled in a generalized progressive hybrid censoring scheme. The maximum likelihood estimators are inferred. In addition, the Bayes estimators are achieved by the Lindley method, as well as the TK method, based on general entropy, squared error and linex loss function, which are all conducted in light of noninformative and informative priors. The performance of the estimates is contrasted according to absolute bias values and mean squared error. The Bayes estimates of proper informative prior are revealed to work better than corresponding noninformative prior estimates. Besides, Bayes estimates produce lower MSEs for two parameters, while for reliability estimation MLEs have lower values instead. A real-life example is also intensively investigated. In the future, more in-depth researches are worth discussing, such as the use of bayesian estimation for the Kumaraswamy distribution in non-linear regression models (Contreras-Reyes et al. [1]).

## Figures and Tables

**Table 1:** The dataset of monthly water capacity of the Shasta reservoir.

0.667157	0.287785	0.126977	0.768563	0.703119	0.729986	0.767135
0.811159	0.829569	0.726164	0.423813	0.715158	0.640395	0.363359
0.463726	0.371904	0.291172	0.414087	0.650691	0.538082	0.744881
0.722613	0.561238	0.813964	0.709025	0.668612	0.524947	0.605979
0.715850	0.529518	0.824860	0.742025	0.468782	0.345075	0.425334
0.767070	0.679829	0.613911	0.461618	0.294834	0.392917	0.688100

**Table 2:** Goodness of different fitted distributions for real data.

Distribution	−ln(L)	AIC	AICc	BIC	K-S	*p*-Value
K(α,β)	−15.6310	−27.2619	−26.9543	−23.7866	0.1905	0.4355
EED	−6.1639	−8.3278	−8.0201	−4.8524	0.2381	0.1859
LD	19.5178	43.0357	43.3434	46.5110	0.3571	0.0089

**Table 3:** The ordered progressive Type-II censored dataset.

0.126977	0.291172	0.345075	0.371904	0.414087	0.425334	0.463726
0.524947	0.538082	0.605979	0.640395	0.667157	0.679829	0.703119
0.715158	0.722613	0.729986	0.744881	0.767135	0.811159	0.824860

**Table 4:** Estimates of α, β and *R* for the real dataset.

Case	Parameter	MLE	SEL	LL		EL		
				*p* = 0.2	*p* = 0.8	*q* = 0.2	*q* = 0.8	
I	α	3.258854	2.844667	1.527920	1.972349	1.768485	1.909330	Lindley
			2.887395	2.739886	2.591068	2.773492	2.714300	TK
	β	2.163197	1.846337	0.194450	0.732673	0.865246	1.012581	Lindley
			1.796699	1.583837	1.465362	1.601074	1.510759	TK
	*R*	0.341358	0.369478	0.493668	0.498473	0.518536	0.537528	Lindley
			0.390156	0.378532	0.376020	0.377140	0.370281	TK
II	α	2.898993	2.556724	1.737647	1.926444	1.848018	1.916395	Lindley
			2.545969	2.400696	2.266208	2.428631	2.367205	TK
	β	1.425335	1.254272	0.521980	0.635828	0.553893	0.640222	Lindley
			1.181996	1.055188	1.000271	1.056085	0.997650	TK
	*R*	0.443953	0.465454	0.536116	0.536250	0.535292	0.534027	Lindley
			0.488082	0.479212	0.476830	0.478343	0.473250	TK
III	α	3.145385	2.781607	2.022479	2.197471	2.110048	2.170816	Lindley
			2.831447	2.696737	2.561594	2.726814	2.672566	TK
	β	1.706230	1.493470	0.751148	0.874202	0.801078	0.881050	Lindley
			1.465403	1.329726	1.258389	1.336265	1.275308	TK
	*R*	0.115402	0.154236	0.226858	0.229274	0.260501	0.287326	Lindley
			0.173029	0.152674	0.150803	0.149099	0.135818	TK

## Appendix A. Simulation Results

**Table A1:** Mean squared errors (MSEs) and absolute biases (ABs) of different estimates of α when T = 0.9.

*n*	*m*	*k*	Sch	MLE	SEL	LL		EL		
						p=0.2	p=0.8	q=0.2	q=0.8	
80	72	20	I	0.195754	0.173274	0.177178	0.183981	0.181016	0.183588	Lindley
				0.342906	0.330704	0.345171	0.355594	0.351718	0.355226	
					0.156018	0.172896	0.173737	0.171160	0.175990	
					0.312790	0.343310	0.349711	0.340308	0.347543	
					0.179724	0.167120	0.172872	0.177911	0.180735	TK
					0.337521	0.326901	0.329848	0.352245	0.354449	
					0.145604	0.150327	0.152585	0.156660	0.163717	
					0.302997	0.314428	0.314487	0.312654	0.320628	
			II	0.184418	0.164741	0.170517	0.189943	0.176538	0.184214	Lindley
				0.334640	0.323862	0.336009	0.359154	0.342129	0.356780	
					0.154296	0.165611	0.173392	0.168059	0.175020	
					0.310359	0.329202	0.344155	0.339885	0.342755	
					0.173730	0.173836	0.174574	0.179825	0.181395	TK
					0.332019	0.327541	0.338772	0.350935	0.352446	
					0.148541	0.152296	0.153598	0.154883	0.155824	
					0.307614	0.311919	0.313886	0.316295	0.319657	
			III	0.186839	0.167715	0.178962	0.182896	0.172983	0.178113	Lindley
				0.331794	0.330235	0.350882	0.358351	0.344087	0.349435	
					0.150382	0.166924	0.172202	0.170060	0.171719	
					0.306341	0.337070	0.345553	0.340878	0.341281	
					0.166749	0.168351	0.168938	0.174207	0.180959	TK
					0.326504	0.330783	0.329393	0.341092	0.351508	
					0.147851	0.148188	0.152516	0.150273	0.153908	
					0.304059	0.309418	0.315851	0.309159	0.314561	
		30	I	0.175359	0.167031	0.172949	0.178008	0.166154	0.173000	Lindley
				0.331946	0.323313	0.339012	0.343564	0.338122	0.344992	
					0.152194	0.164958	0.169652	0.166430	0.168725	
					0.310347	0.338482	0.343621	0.335488	0.339530	
					0.166400	0.166638	0.172391	0.171438	0.177878	TK
					0.320847	0.324453	0.333654	0.340759	0.344098	
					0.143990	0.149207	0.150038	0.153138	0.155757	
					0.305919	0.312988	0.320139	0.317226	0.318324	
			II	0.180962	0.161311	0.163980	0.181175	0.174875	0.181670	Lindley
				0.330292	0.322350	0.329987	0.352216	0.344300	0.349224	
					0.152342	0.161924	0.167277	0.162367	0.165248	
					0.310690	0.333965	0.336807	0.332194	0.337640	
					0.168375	0.160220	0.162163	0.172300	0.176658	TK
					0.326550	0.321193	0.326577	0.343392	0.344013	
					0.148364	0.150361	0.151663	0.151177	0.153493	
					0.308773	0.312301	0.313243	0.314156	0.316487	
			III	0.185089	0.165199	0.168558	0.177434	0.170770	0.175226	Lindley
				0.338752	0.319409	0.339189	0.350066	0.341316	0.342927	
					0.144593	0.165095	0.170556	0.164992	0.170073	
					0.303143	0.336506	0.343016	0.336226	0.342143	
					0.165939	0.157391	0.158262	0.168973	0.171244	TK
					0.324860	0.319667	0.325512	0.338378	0.339089	
					0.141980	0.142420	0.151584	0.148092	0.153502	
					0.300193	0.303745	0.314158	0.309872	0.312044	
	76	20	I	0.166300	0.162560	0.167433	0.176058	0.173686	0.177993	Lindley
				0.317472	0.326134	0.336984	0.348843	0.345815	0.348391	
					0.147973	0.158480	0.161429	0.163395	0.166470	
					0.303859	0.329001	0.328622	0.330609	0.334530	
					0.165071	0.158974	0.161826	0.170886	0.177120	TK
					0.324415	0.321772	0.325416	0.336610	0.352839	
					0.142796	0.143406	0.147027	0.149405	0.151933	
					0.301857	0.306838	0.311106	0.308166	0.316401	
			II	0.171547	0.161124	0.163368	0.173401	0.171781	0.174262	Lindley
				0.327783	0.322533	0.329479	0.342409	0.341883	0.348672	
					0.150441	0.156468	0.163099	0.156584	0.160757	
					0.315480	0.328370	0.333625	0.323330	0.334570	
					0.162611	0.150112	0.157015	0.169074	0.172704	TK
					0.319556	0.312856	0.322624	0.338347	0.341468	
					0.142540	0.146455	0.149506	0.146997	0.152814	
					0.299316	0.308749	0.314981	0.306933	0.309555	
			III	0.170692	0.159287	0.168563	0.173856	0.169945	0.172634	Lindley
				0.323645	0.317932	0.339618	0.346388	0.334563	0.341489	
					0.142341	0.154256	0.165134	0.158654	0.162581	
					0.299201	0.327005	0.333447	0.326716	0.333624	
					0.161554	0.151212	0.157969	0.166336	0.169676	TK
					0.313544	0.313482	0.319093	0.335268	0.336643	
					0.136250	0.140744	0.148001	0.139521	0.143761	
					0.292927	0.298367	0.317634	0.295863	0.304371	
		30	I	0.165008	0.155958	0.166295	0.173885	0.166357	0.167108	Lindley
				0.317973	0.317333	0.335355	0.347527	0.333399	0.337807	
					0.143072	0.154050	0.161061	0.162549	0.164274	
					0.301736	0.317248	0.333638	0.334190	0.337059	
					0.159416	0.157680	0.159625	0.166577	0.174166	TK
					0.315904	0.318598	0.322230	0.336044	0.351277	
					0.133193	0.136237	0.140467	0.146210	0.148110	
					0.289755	0.297556	0.304069	0.306296	0.309866	
			II	0.163385	0.160461	0.161430	0.173194	0.167663	0.171512	Lindley
				0.323427	0.315128	0.321918	0.344371	0.330463	0.343644	
					0.145763	0.150587	0.161033	0.156015	0.158450	
					0.306658	0.322662	0.333448	0.328242	0.325840	
					0.157694	0.146225	0.156718	0.167753	0.171009	TK
					0.311193	0.303677	0.319265	0.337101	0.341809	
					0.141954	0.141954	0.146235	0.144139	0.149823	
					0.302460	0.302218	0.306650	0.301372	0.308069	
			III	0.169881	0.155722	0.162502	0.168595	0.158510	0.166633	Lindley
				0.328002	0.315869	0.332490	0.336967	0.328002	0.332975	
					0.140030	0.151151	0.163956	0.157448	0.158148	
					0.304585	0.320872	0.335253	0.328741	0.329517	
					0.152195	0.146831	0.153871	0.159082	0.164530	TK
					0.310705	0.302980	0.315690	0.332910	0.333448	
					0.133141	0.136999	0.145394	0.137570	0.141221	
					0.292911	0.296466	0.305983	0.295833	0.304130	
100	82	30	I	0.158496	0.147905	0.151299	0.157098	0.156028	0.158770	Lindley
				0.316708	0.302370	0.315620	0.328098	0.318789	0.327788	
					0.133493	0.146950	0.156503	0.148382	0.149195	
					0.284853	0.316906	0.325588	0.316339	0.317492	
					0.143107	0.136020	0.140488	0.148848	0.154655	TK
					0.303305	0.287868	0.304398	0.315195	0.324310	
					0.127538	0.128523	0.130793	0.133431	0.136239	
					0.286952	0.290597	0.296908	0.288247	0.295697	
			II	0.160541	0.136889	0.148014	0.150984	0.142043	0.150085	Lindley
				0.311813	0.293170	0.314510	0.320854	0.311251	0.321959	
					0.120695	0.133282	0.143825	0.138859	0.148270	
					0.280028	0.301934	0.309866	0.309463	0.320493	
					0.135606	0.135565	0.139901	0.142889	0.149675	TK
					0.291778	0.294604	0.301028	0.312668	0.321747	
					0.120401	0.121388	0.129463	0.128332	0.137610	
					0.276503	0.278955	0.290272	0.283403	0.295037	
			III	0.161975	0.139471	0.148770	0.152676	0.141963	0.154038	Lindley
				0.316543	0.300236	0.319234	0.324589	0.309442	0.320746	
					0.132432	0.148393	0.154412	0.147061	0.153666	
					0.287758	0.320192	0.327141	0.318151	0.324176	
					0.147390	0.136199	0.141653	0.154729	0.163258	TK
					0.304159	0.292840	0.299760	0.328307	0.336531	
					0.121536	0.123586	0.129319	0.122734	0.128961	
					0.277521	0.281209	0.289905	0.280488	0.289221	
		45	I	0.156002	0.139135	0.145639	0.152580	0.153124	0.156099	Lindley
				0.310401	0.296142	0.310395	0.322577	0.318429	0.325840	
					0.130348	0.142467	0.148427	0.141910	0.143547	
					0.287030	0.312038	0.315099	0.309775	0.311625	
					0.136699	0.131594	0.139590	0.148293	0.149381	TK
					0.296439	0.289784	0.306626	0.317400	0.315475	
					0.121002	0.124990	0.129255	0.127068	0.130692	
					0.281013	0.282024	0.291731	0.291606	0.290489	
			II	0.147847	0.134692	0.142668	0.145524	0.135065	0.147970	Lindley
				0.301514	0.291427	0.307242	0.317248	0.300149	0.318908	
					0.119197	0.125736	0.135094	0.135443	0.142335	
					0.274329	0.292137	0.302134	0.303815	0.310954	
					0.134948	0.128939	0.131073	0.140155	0.141756	TK
					0.295057	0.283383	0.293255	0.310308	0.310028	
					0.118239	0.119540	0.121986	0.125353	0.126930	
					0.271242	0.279481	0.280613	0.284735	0.285185	
			III	0.147374	0.131965	0.146877	0.157459	0.140713	0.145988	Lindley
				0.303809	0.290647	0.317034	0.329717	0.309025	0.316835	
					0.125244	0.144764	0.153404	0.143873	0.148504	
					0.282434	0.316083	0.323914	0.310676	0.318113	
					0.139522	0.133469	0.140116	0.153783	0.156486	TK
					0.295972	0.290425	0.301687	0.326263	0.330464	
					0.119613	0.121515	0.122695	0.122891	0.124598	
					0.277887	0.280573	0.284917	0.283618	0.284538	
	94	30	I	0.132503	0.123797	0.143583	0.144992	0.144589	0.136430	Lindley
				0.285001	0.284999	0.309513	0.312495	0.309185	0.302944	
					0.120096	0.124511	0.132111	0.123763	0.130444	
					0.273884	0.288897	0.298815	0.283869	0.298420	
					0.126725	0.130660	0.133471	0.139268	0.148050	TK
					0.280512	0.295638	0.292624	0.304014	0.313696	
					0.114178	0.115188	0.121156	0.117458	0.118981	
					0.266358	0.275687	0.277339	0.276102	0.272325	
			II	0.135851	0.124772	0.135698	0.142222	0.129342	0.132362	Lindley
				0.292391	0.285486	0.301356	0.310467	0.294303	0.293817	
					0.115023	0.120829	0.128950	0.132106	0.139177	
					0.261426	0.283954	0.292267	0.299527	0.307270	
					0.132854	0.126923	0.128081	0.132956	0.137500	TK
					0.291374	0.284487	0.291459	0.294028	0.303618	
					0.113034	0.116862	0.120460	0.118538	0.121378	
					0.267837	0.272753	0.281879	0.277129	0.280367	
			III	0.130014	0.129753	0.133493	0.139957	0.131939	0.132621	Lindley
				0.280600	0.284624	0.297418	0.307268	0.295253	0.298255	
					0.123672	0.123614	0.137455	0.124610	0.130591	
					0.281759	0.287829	0.304745	0.291429	0.294189	
					0.123487	0.127427	0.132597	0.131822	0.133141	TK
					0.278401	0.285310	0.289593	0.298348	0.299885	
					0.114975	0.115972	0.120293	0.119745	0.121723	
					0.267577	0.272509	0.281483	0.276880	0.281057	
		45	I	0.129608	0.121916	0.127324	0.137703	0.135786	0.139453	Lindley
				0.285412	0.275237	0.291390	0.305126	0.300680	0.306916	
					0.114474	0.120666	0.128696	0.115819	0.123577	
					0.271550	0.288897	0.295148	0.276665	0.285389	
					0.121648	0.122535	0.124250	0.135400	0.142578	TK
					0.274330	0.278488	0.284925	0.303517	0.311050	
					0.108882	0.111858	0.114731	0.116679	0.117557	
					0.265101	0.269715	0.271830	0.275539	0.274517	
			II	0.130496	0.123406	0.130476	0.138231	0.124809	0.130446	Lindley
				0.283334	0.281610	0.296144	0.304885	0.289933	0.294102	
					0.111379	0.119155	0.125578	0.129603	0.133119	
					0.266771	0.281377	0.288220	0.298622	0.303524	
					0.126575	0.122176	0.127958	0.129204	0.132621	TK
					0.278567	0.280139	0.287326	0.293037	0.296470	
					0.109182	0.112205	0.115913	0.117274	0.120175	
					0.263443	0.270690	0.271220	0.273281	0.274126	
			III	0.131854	0.120313	0.126645	0.136001	0.127302	0.127935	Lindley
				0.291057	0.275473	0.293714	0.307007	0.292345	0.292044	
					0.111521	0.120994	0.125653	0.123463	0.126743	
					0.265186	0.284167	0.291224	0.285998	0.291452	
					0.121873	0.121860	0.124848	0.126331	0.129101	TK
					0.280869	0.282387	0.284658	0.286454	0.293051	
					0.113572	0.113962	0.114960	0.113353	0.118752	
					0.269484	0.267684	0.271999	0.265857	0.278690	

**Table A2:** MSEs and ABs of different estimates of β when T = 0.9.

*n*	*m*	*k*	Sch	MLE	SEL	LL		EL		
						p=0.2	p=0.8	q=0.2	q=0.8	
80	72	20	I	0.195961	0.167474	0.125922	0.129332	0.170324	0.179334	Lindley
				0.336624	0.316846	0.295114	0.295521	0.345211	0.356905	
					0.151557	0.112942	0.125658	0.162986	0.168371	
					0.299841	0.280949	0.295638	0.335910	0.341832	
					0.161133	0.143960	0.147142	0.154170	0.155740	TK
					0.312385	0.301201	0.306155	0.310556	0.315511	
					0.146008	0.130093	0.131761	0.134778	0.147166	
					0.295375	0.285622	0.290624	0.293504	0.302999	
			II	0.198616	0.169368	0.118020	0.122983	0.163528	0.169168	Lindley
				0.332629	0.313657	0.286155	0.288221	0.336406	0.344660	
					0.144250	0.110326	0.121125	0.158115	0.166199	
					0.296263	0.271621	0.291258	0.331832	0.340948	
					0.160701	0.145519	0.147751	0.146622	0.151650	TK
					0.307795	0.296878	0.302154	0.295988	0.312282	
					0.143657	0.125874	0.127691	0.131021	0.136678	
					0.295470	0.279471	0.286267	0.289794	0.294870	
			III	0.199714	0.163990	0.114182	0.125746	0.160421	0.165573	Lindley
				0.334281	0.311947	0.280464	0.299706	0.330428	0.342646	
					0.155875	0.109529	0.116644	0.155576	0.161524	
					0.303372	0.276485	0.285201	0.331001	0.339624	
					0.169871	0.150367	0.158423	0.168751	0.171984	TK
					0.315943	0.303936	0.307854	0.310915	0.323535	
					0.161258	0.128393	0.129483	0.138433	0.140673	
					0.308045	0.283221	0.284467	0.293484	0.290108	
		30	I	0.180081	0.164770	0.127594	0.127918	0.164446	0.171331	Lindley
				0.326501	0.321166	0.295031	0.296843	0.340540	0.339008	
					0.148262	0.111336	0.117181	0.161642	0.162909	
					0.296860	0.276169	0.284330	0.335575	0.336046	
					0.160261	0.142307	0.148250	0.146628	0.149070	TK
					0.312541	0.304625	0.305431	0.303011	0.308049	
					0.145959	0.122722	0.127027	0.126777	0.131688	
					0.296422	0.282862	0.286921	0.280670	0.289439	
			II	0.194645	0.157488	0.115380	0.119628	0.158545	0.168903	Lindley
				0.330164	0.308937	0.276924	0.289372	0.329407	0.343435	
					0.136508	0.108672	0.114623	0.152909	0.160779	
					0.287950	0.269303	0.285936	0.323521	0.333769	
					0.159316	0.136843	0.137329	0.141616	0.150826	TK
					0.307774	0.294194	0.294585	0.297899	0.299668	
					0.137508	0.121449	0.124589	0.128433	0.132614	
					0.290293	0.278860	0.285039	0.287289	0.286509	
			III	0.185316	0.159978	0.110708	0.119849	0.158934	0.161440	Lindley
				0.326110	0.304847	0.277643	0.290884	0.330500	0.336606	
					0.145412	0.105190	0.112186	0.154588	0.157105	
					0.293478	0.268608	0.276084	0.328198	0.333355	
					0.156214	0.141367	0.151079	0.149774	0.155430	TK
					0.305781	0.294312	0.303219	0.303343	0.306630	
					0.146418	0.127305	0.128522	0.136888	0.139216	
					0.290897	0.286155	0.285302	0.290073	0.299831	
	76	20	I	0.166291	0.156298	0.118440	0.124711	0.163635	0.165813	Lindley
				0.313605	0.309953	0.284341	0.293997	0.337621	0.339916	
					0.141413	0.106678	0.112972	0.156046	0.159913	
					0.290861	0.270763	0.277584	0.329064	0.331369	
					0.159837	0.137589	0.144562	0.144789	0.145483	TK
					0.307406	0.290110	0.304950	0.297334	0.303843	
					0.134403	0.120996	0.124788	0.125574	0.127552	
					0.295813	0.280510	0.277648	0.286978	0.282143	
			II	0.171110	0.157852	0.113267	0.115085	0.159968	0.163557	Lindley
				0.312218	0.302511	0.276817	0.278177	0.333592	0.335084	
					0.133413	0.103128	0.106524	0.150006	0.153817	
					0.283053	0.264280	0.269033	0.325811	0.326850	
					0.158836	0.128150	0.135009	0.133405	0.145423	TK
					0.303372	0.282541	0.295419	0.285841	0.296925	
					0.129618	0.119702	0.122264	0.123452	0.128127	
					0.280295	0.277571	0.279210	0.280092	0.285718	
			III	0.173008	0.151509	0.111720	0.113288	0.154259	0.156918	Lindley
				0.315390	0.298115	0.271731	0.277697	0.326029	0.330195	
					0.134863	0.104234	0.111153	0.148866	0.153811	
					0.279596	0.268118	0.277566	0.319942	0.321904	
					0.147908	0.126286	0.131559	0.143254	0.153825	TK
					0.298540	0.278969	0.290751	0.292373	0.310053	
					0.137544	0.120727	0.123537	0.123710	0.126293	
					0.284469	0.273705	0.279740	0.280318	0.285400	
		30	I	0.161044	0.153009	0.116763	0.119038	0.159944	0.162030	Lindley
				0.311460	0.301180	0.281434	0.284181	0.330128	0.333800	
					0.135199	0.102043	0.111867	0.154140	0.159295	
					0.293265	0.265156	0.275608	0.328299	0.332644	
					0.158301	0.136716	0.141035	0.142355	0.145560	TK
					0.303930	0.292881	0.302139	0.297320	0.304800	
					0.133585	0.115898	0.123867	0.123895	0.124198	
					0.287951	0.272650	0.285000	0.282525	0.286325	
			II	0.161795	0.153771	0.111200	0.114567	0.158889	0.161932	Lindley
				0.312143	0.299332	0.272651	0.279835	0.330389	0.333344	
					0.130638	0.101022	0.105920	0.144542	0.151853	
					0.281231	0.262691	0.268349	0.310455	0.321634	
					0.153926	0.127973	0.129349	0.129577	0.137293	TK
					0.303576	0.287272	0.290564	0.280717	0.292569	
					0.128546	0.115852	0.119988	0.122094	0.127185	
					0.276583	0.271327	0.278617	0.279357	0.287922	
			III	0.161182	0.151934	0.107491	0.113610	0.152346	0.155006	Lindley
				0.301764	0.299076	0.269735	0.278473	0.322370	0.326885	
					0.129530	0.103948	0.106640	0.140239	0.147796	
					0.281968	0.265223	0.272102	0.309017	0.320355	
					0.146521	0.125052	0.133339	0.136219	0.151772	TK
					0.301641	0.285029	0.291321	0.291023	0.301340	
					0.134788	0.118289	0.123377	0.120359	0.124732	
					0.282284	0.277166	0.285947	0.269854	0.281490	
100	82	30	I	0.153310	0.145301	0.109663	0.116788	0.149552	0.156306	Lindley
				0.299369	0.296725	0.274491	0.281803	0.321317	0.328219	
					0.129155	0.099731	0.108994	0.146049	0.147860	
					0.279922	0.262859	0.275537	0.314855	0.315498	
					0.147623	0.126485	0.130951	0.128531	0.131542	TK
					0.292822	0.283461	0.288492	0.288371	0.290200	
					0.125685	0.115185	0.118393	0.117438	0.121412	
					0.278130	0.270371	0.277522	0.271197	0.281743	
			II	0.154574	0.140626	0.100325	0.108500	0.144246	0.145797	Lindley
				0.299583	0.283391	0.263969	0.273716	0.313823	0.315593	
					0.127260	0.096169	0.101876	0.135626	0.140571	
					0.275750	0.258034	0.263246	0.308420	0.314007	
					0.138403	0.122640	0.125227	0.127564	0.128041	TK
					0.287158	0.276862	0.279997	0.281649	0.283290	
					0.124515	0.114230	0.116840	0.121398	0.126292	
					0.277029	0.266471	0.277677	0.277725	0.278306	
			III	0.167441	0.142744	0.104775	0.108659	0.149072	0.154847	Lindley
				0.307912	0.289845	0.271904	0.276128	0.326706	0.333213	
					0.125183	0.096211	0.104054	0.138582	0.142786	
					0.273730	0.254977	0.268693	0.312299	0.321215	
					0.145117	0.116617	0.122610	0.129743	0.136671	TK
					0.288590	0.267895	0.280975	0.284886	0.297167	
					0.132132	0.117712	0.122337	0.118227	0.125430	
					0.282436	0.261177	0.278052	0.268608	0.283806	
		45	I	0.143500	0.136130	0.102379	0.112711	0.145897	0.151619	Lindley
				0.294335	0.295757	0.262240	0.277892	0.314799	0.322754	
					0.120029	0.096214	0.106596	0.141639	0.147179	
					0.270996	0.258052	0.272952	0.315186	0.316920	
					0.137385	0.125236	0.128988	0.123161	0.125961	TK
					0.292469	0.279982	0.283197	0.273377	0.280845	
					0.121384	0.112881	0.114619	0.112884	0.117658	
					0.272890	0.265296	0.270659	0.265413	0.273428	
			II	0.149713	0.137068	0.100365	0.104634	0.135397	0.139903	Lindley
				0.296191	0.289884	0.258184	0.267517	0.300554	0.313164	
					0.125390	0.088489	0.100022	0.129176	0.137732	
					0.273556	0.245052	0.263698	0.296608	0.308541	
					0.134871	0.116786	0.123637	0.124978	0.127771	TK
					0.283711	0.270975	0.282927	0.277271	0.283818	
					0.123139	0.111609	0.113592	0.117852	0.123638	
					0.270458	0.265015	0.271183	0.266431	0.277810	
			III	0.162169	0.140610	0.103915	0.109819	0.143020	0.151550	Lindley
				0.306268	0.288011	0.265563	0.279020	0.322923	0.326686	
					0.123127	0.094063	0.097745	0.137214	0.140252	
					0.274663	0.253412	0.260985	0.306914	0.318428	
					0.127541	0.116658	0.119159	0.128263	0.135881	TK
					0.278944	0.265494	0.278574	0.288084	0.290577	
					0.124970	0.111977	0.119678	0.118157	0.123543	
					0.278475	0.269041	0.276553	0.269595	0.277491	
	94	30	I	0.145292	0.128537	0.101165	0.103913	0.130634	0.144583	Lindley
				0.295310	0.278937	0.260241	0.267477	0.301540	0.315142	
					0.116543	0.087605	0.093752	0.121408	0.125169	
					0.265539	0.241199	0.253960	0.288968	0.292778	
					0.126814	0.111004	0.119686	0.115347	0.117204	TK
					0.273766	0.262243	0.272892	0.266599	0.270314	
					0.115467	0.100148	0.103070	0.106509	0.107580	
					0.268378	0.252260	0.258183	0.260890	0.262149	
			II	0.135628	0.121336	0.094265	0.098833	0.124486	0.130945	Lindley
				0.278024	0.271159	0.254310	0.256069	0.290725	0.301405	
					0.116163	0.082077	0.094949	0.118768	0.123681	
					0.265434	0.231296	0.255256	0.285416	0.291467	
					0.122309	0.113803	0.122946	0.109296	0.111378	TK
					0.273799	0.266610	0.276905	0.261515	0.267054	
					0.110301	0.103668	0.105035	0.103877	0.109838	
					0.258339	0.254095	0.258928	0.258267	0.262114	
			III	0.137106	0.126933	0.088083	0.091224	0.130196	0.133326	Lindley
				0.279503	0.273917	0.244921	0.245288	0.297581	0.306710	
					0.106781	0.085160	0.089138	0.116780	0.123435	
					0.254896	0.239704	0.247836	0.279106	0.293215	
					0.121616	0.112660	0.113223	0.109145	0.115564	TK
					0.270790	0.266725	0.266141	0.262436	0.265160	
					0.116904	0.106376	0.107909	0.112116	0.113828	
					0.267235	0.256490	0.257001	0.258203	0.260865	
		45	I	0.138448	0.107788	0.097626	0.101991	0.126968	0.134152	Lindley
				0.277945	0.250081	0.256038	0.263544	0.293315	0.298622	
					0.109824	0.084894	0.091513	0.120331	0.121207	
					0.258606	0.240529	0.247569	0.287617	0.291623	
					0.118166	0.108389	0.111368	0.109802	0.113726	TK
					0.268184	0.263430	0.269003	0.258699	0.274274	
					0.092468	0.096069	0.101331	0.099836	0.105263	
					0.242037	0.247773	0.254265	0.256141	0.256763	
			II	0.132378	0.109593	0.092566	0.099696	0.114651	0.127986	Lindley
				0.280257	0.266184	0.246536	0.259982	0.277877	0.297383	
					0.102115	0.080137	0.087635	0.116177	0.117395	
					0.251239	0.232838	0.242800	0.277206	0.280823	
					0.116214	0.108690	0.111429	0.106700	0.108722	TK
					0.264074	0.259276	0.266296	0.258925	0.260910	
					0.098265	0.092258	0.098959	0.102296	0.107582	
					0.248720	0.241530	0.254006	0.248100	0.258353	
			III	0.128764	0.106079	0.087320	0.088729	0.118343	0.121471	Lindley
				0.275581	0.253820	0.241452	0.240844	0.280548	0.288014	
					0.104581	0.083902	0.088412	0.110973	0.119573	
					0.251543	0.236843	0.246940	0.278984	0.285724	
					0.109023	0.109590	0.112305	0.114869	0.112612	TK
					0.255856	0.257675	0.270349	0.268982	0.268540	
					0.105243	0.104414	0.105186	0.107633	0.109181	
					0.252713	0.254990	0.259515	0.257580	0.262889	

**Table A3:** MSEs and ABs of different estimates of R(t) when T = 0.9.

*n*	*m*	*k*	Sch	MLE	SEL	LL		EL		
						p=0.2	p=0.8	q=0.2	q=0.8	
80	72	20	I	0.000680	0.000732	0.000948	0.000929	0.000805	0.000747	Lindley
				0.021221	0.021300	0.023400	0.023693	0.021764	0.021364	
					0.000661	0.000910	0.000870	0.000760	0.000709	
					0.020059	0.023549	0.022733	0.021204	0.020385	
					0.000936	0.000786	0.000758	0.000765	0.000728	TK
					0.023562	0.021801	0.021501	0.021273	0.020447	
					0.000872	0.000722	0.000705	0.000690	0.000678	
					0.023000	0.021268	0.020911	0.020531	0.020289	
			II	0.000675	0.000715	0.000956	0.000924	0.000766	0.000712	Lindley
				0.020644	0.020700	0.023929	0.023428	0.021223	0.020658	
					0.000689	0.000831	0.000819	0.000683	0.000621	
					0.020402	0.022467	0.021821	0.020360	0.019234	
					0.000870	0.000728	0.000724	0.000742	0.000701	TK
					0.022784	0.021290	0.020705	0.020560	0.020320	
					0.000775	0.000693	0.000689	0.000680	0.000673	
					0.021775	0.020597	0.020647	0.020372	0.020930	
			III	0.000665	0.000702	0.000869	0.000783	0.000737	0.000711	Lindley
				0.020451	0.020831	0.022992	0.022209	0.020881	0.020408	
					0.000666	0.000815	0.000777	0.000736	0.000668	
					0.020126	0.022329	0.021622	0.020929	0.019818	
					0.000801	0.000714	0.000683	0.000780	0.000737	TK
					0.022231	0.020929	0.020802	0.021767	0.021373	
					0.000781	0.000662	0.000654	0.000731	0.000671	
					0.021906	0.020212	0.020332	0.021428	0.020791	
		30	I	0.000677	0.000725	0.000924	0.000902	0.000771	0.000696	Lindley
				0.020729	0.020828	0.023448	0.023393	0.021321	0.020477	
					0.000652	0.000904	0.000828	0.000745	0.000638	
					0.019674	0.023443	0.022439	0.020989	0.018967	
					0.000860	0.000776	0.000753	0.000717	0.000681	TK
					0.022688	0.021543	0.021578	0.020461	0.019850	
					0.000822	0.000705	0.000683	0.000688	0.000635	
					0.022004	0.020932	0.020610	0.020710	0.019971	
			II	0.000673	0.000709	0.000921	0.000902	0.000762	0.000701	Lindley
				0.020854	0.020803	0.023353	0.023191	0.021567	0.020409	
					0.000675	0.000814	0.000803	0.000648	0.000613	
					0.020321	0.022145	0.022206	0.019319	0.019369	
					0.000789	0.000709	0.000696	0.000733	0.000673	TK
					0.021696	0.020700	0.020576	0.020667	0.020036	
					0.000747	0.000683	0.000665	0.000666	0.000658	
					0.021355	0.020473	0.020097	0.020487	0.020202	
			III	0.000660	0.000701	0.000863	0.000786	0.000705	0.000694	Lindley
				0.020289	0.020702	0.022739	0.021965	0.020427	0.020374	
					0.000645	0.000800	0.000776	0.000667	0.000649	
					0.019935	0.022084	0.021785	0.019299	0.018283	
					0.000761	0.000695	0.000651	0.000729	0.000698	TK
					0.021680	0.020592	0.019512	0.021466	0.021139	
					0.000713	0.000658	0.000649	0.000682	0.000650	
					0.021065	0.020173	0.019905	0.020716	0.020204	
	76	20	I	0.000653	0.000694	0.000904	0.000832	0.000768	0.000655	Lindley
				0.020315	0.021077	0.023445	0.022338	0.021549	0.020125	
					0.000634	0.000840	0.000824	0.000709	0.000611	
					0.019584	0.022508	0.022294	0.020615	0.018883	
					0.000782	0.000727	0.000697	0.000702	0.000671	TK
					0.022318	0.021386	0.020711	0.020748	0.019608	
					0.000801	0.000666	0.000639	0.000667	0.000626	
					0.022149	0.020431	0.019431	0.020438	0.019762	
			II	0.000644	0.000705	0.000855	0.000802	0.000725	0.000682	Lindley
				0.019746	0.021068	0.022487	0.022045	0.020959	0.020161	
					0.000641	0.000768	0.000757	0.000636	0.000610	
					0.019607	0.021413	0.021487	0.019237	0.019280	
					0.000770	0.000681	0.000678	0.000724	0.000653	TK
					0.021766	0.020400	0.020485	0.020478	0.019413	
					0.000738	0.000667	0.000641	0.000652	0.000623	
					0.021486	0.020214	0.020009	0.020327	0.019468	
			III	0.000621	0.000671	0.000847	0.000752	0.000693	0.000680	Lindley
				0.019875	0.019882	0.022530	0.021327	0.020294	0.020042	
					0.000635	0.000776	0.000740	0.000665	0.000594	
					0.019621	0.021799	0.021147	0.020100	0.018962	
					0.000749	0.000699	0.000678	0.000687	0.000653	TK
					0.021223	0.020906	0.020528	0.020929	0.020301	
					0.000708	0.000646	0.000641	0.000672	0.000638	
					0.020335	0.020315	0.019949	0.020411	0.019931	
		30	I	0.000615	0.000636	0.000880	0.000843	0.000732	0.000634	Lindley
				0.019902	0.019840	0.023224	0.022258	0.020581	0.019276	
					0.000631	0.000806	0.000761	0.000676	0.000605	
					0.019871	0.022081	0.021549	0.019964	0.019266	
					0.000757	0.000723	0.000667	0.000681	0.000648	TK
					0.021585	0.021283	0.019895	0.020203	0.019780	
					0.000786	0.000644	0.000620	0.000643	0.000605	
					0.021498	0.019961	0.019965	0.019987	0.019543	
			II	0.000615	0.000692	0.000841	0.000815	0.000675	0.000647	Lindley
				0.019846	0.020606	0.022456	0.022072	0.020072	0.019722	
					0.000627	0.000755	0.000746	0.000612	0.000591	
					0.019496	0.021489	0.021290	0.019024	0.018930	
					0.000766	0.000674	0.000671	0.000708	0.000636	TK
					0.021311	0.020548	0.020687	0.020480	0.019429	
					0.000694	0.000660	0.000617	0.000632	0.000615	
					0.020344	0.020437	0.019428	0.019844	0.019475	
			III	0.000611	0.000635	0.000801	0.000791	0.000684	0.000634	Lindley
				0.019298	0.019770	0.021973	0.021822	0.020058	0.019568	
					0.000616	0.000746	0.000738	0.000617	0.000584	
					0.019645	0.021032	0.020939	0.019341	0.018644	
					0.000729	0.000661	0.000656	0.000682	0.000646	TK
					0.021096	0.020132	0.019980	0.020641	0.020678	
					0.000665	0.000623	0.000614	0.000643	0.000621	
					0.020242	0.019498	0.019681	0.019857	0.019696	
100	82	30	I	0.000570	0.000626	0.000816	0.000795	0.000663	0.000606	Lindley
				0.019244	0.019619	0.022522	0.022213	0.019943	0.019300	
					0.000613	0.000787	0.000748	0.000637	0.000554	
					0.019475	0.021835	0.021419	0.019586	0.018017	
					0.000702	0.000659	0.000640	0.000659	0.000622	TK
					0.020832	0.020162	0.019371	0.019672	0.019578	
					0.000688	0.000630	0.000609	0.000635	0.000581	
					0.020424	0.019536	0.019674	0.019614	0.019052	
			II	0.000575	0.000673	0.000751	0.000716	0.000639	0.000604	Lindley
				0.019300	0.020583	0.021177	0.020981	0.019689	0.019076	
					0.000612	0.000718	0.000715	0.000589	0.000545	
					0.019590	0.020796	0.020832	0.018479	0.018247	
					0.000624	0.000646	0.000594	0.000602	0.000548	TK
					0.019774	0.020069	0.018964	0.019290	0.018183	
					0.000622	0.000597	0.000562	0.000581	0.000521	
					0.019444	0.019335	0.018876	0.018947	0.018310	
			III	0.000566	0.000621	0.000801	0.000782	0.000676	0.000631	Lindley
				0.018845	0.020090	0.022612	0.022132	0.019984	0.019142	
					0.000610	0.000744	0.000736	0.000637	0.000557	
					0.019361	0.021549	0.021670	0.019870	0.017859	
					0.000658	0.000655	0.000620	0.000659	0.000634	TK
					0.020421	0.020155	0.020033	0.020108	0.019788	
					0.000639	0.000602	0.000601	0.000627	0.000608	
					0.019989	0.019290	0.019432	0.019942	0.019825	
		45	I	0.000550	0.000592	0.000795	0.000786	0.000631	0.000599	Lindley
				0.018817	0.019087	0.022107	0.021457	0.019449	0.018992	
					0.000595	0.000770	0.000768	0.000616	0.000530	
					0.019444	0.021324	0.021347	0.019236	0.017887	
					0.000661	0.000651	0.000622	0.000644	0.000613	TK
					0.019928	0.020014	0.019453	0.019574	0.019078	
					0.000658	0.000612	0.000604	0.000582	0.000569	
					0.019910	0.019707	0.019247	0.019064	0.019261	
			II	0.000523	0.000671	0.000737	0.000714	0.000631	0.000595	Lindley
				0.018444	0.019915	0.020792	0.020860	0.019666	0.019158	
					0.000581	0.000715	0.000709	0.000561	0.000528	
					0.019226	0.020832	0.020940	0.018287	0.017826	
					0.000589	0.000598	0.000591	0.000598	0.000585	TK
					0.019083	0.019322	0.019312	0.019011	0.018811	
					0.000574	0.000578	0.000511	0.000578	0.000511	
					0.018596	0.019114	0.017982	0.019206	0.018079	
			III	0.000539	0.000618	0.000783	0.000757	0.000663	0.000615	Lindley
				0.018597	0.019525	0.022204	0.021531	0.019797	0.019171	
					0.000597	0.000736	0.000731	0.000635	0.000542	
					0.019321	0.021223	0.021196	0.019090	0.017538	
					0.000650	0.000645	0.000617	0.000642	0.000610	TK
					0.020041	0.020339	0.019763	0.019904	0.019756	
					0.000631	0.000579	0.000570	0.000609	0.000602	
					0.019749	0.019151	0.018960	0.019855	0.019514	
	94	30	I	0.000522	0.000545	0.000692	0.000668	0.000562	0.000547	Lindley
				0.018529	0.018453	0.020509	0.020163	0.018294	0.018571	
					0.000531	0.000650	0.000626	0.000548	0.000527	
					0.017891	0.020160	0.019099	0.018346	0.017668	
					0.000640	0.000618	0.000602	0.000623	0.000490	TK
					0.019697	0.019483	0.019470	0.019186	0.017301	
					0.000609	0.000567	0.000554	0.000562	0.000506	
					0.019095	0.018696	0.018325	0.018657	0.017774	
			II	0.000482	0.000577	0.000673	0.000661	0.000608	0.000586	Lindley
				0.017448	0.018630	0.019999	0.019995	0.019440	0.018809	
					0.000528	0.000645	0.000638	0.000552	0.000505	
					0.018033	0.019869	0.019285	0.018197	0.017324	
					0.000580	0.000558	0.000518	0.000569	0.000545	TK
					0.018751	0.018459	0.017972	0.018244	0.017945	
					0.000555	0.000518	0.000493	0.000553	0.000491	
					0.018425	0.018058	0.017274	0.018342	0.017762	
			III	0.000507	0.000539	0.000636	0.000619	0.000583	0.000561	Lindley
				0.017852	0.018343	0.019598	0.019043	0.018788	0.018717	
					0.000524	0.000610	0.000592	0.000549	0.000487	
					0.018098	0.019172	0.018937	0.017739	0.017465	
					0.000567	0.000546	0.000521	0.000571	0.000563	TK
					0.018663	0.018532	0.018198	0.018791	0.018845	
					0.000547	0.000530	0.000508	0.000533	0.000521	
					0.018295	0.018022	0.017656	0.018278	0.017970	
		45	I	0.000489	0.000518	0.000665	0.000643	0.000532	0.000469	Lindley
				0.017770	0.017943	0.020333	0.019843	0.017780	0.016934	
					0.000494	0.000641	0.000608	0.000517	0.000485	
					0.017468	0.019771	0.019158	0.017413	0.017298	
					0.000580	0.000571	0.000561	0.000564	0.000479	TK
					0.018467	0.018827	0.018295	0.018545	0.017103	
					0.000555	0.000528	0.000499	0.000542	0.000482	
					0.018141	0.018012	0.017684	0.018459	0.017565	
			II	0.000473	0.000513	0.000626	0.000582	0.000576	0.000501	Lindley
				0.017216	0.017991	0.019267	0.018671	0.018518	0.017220	
					0.000510	0.000612	0.000552	0.000523	0.000460	
					0.017570	0.019417	0.018322	0.018026	0.016693	
					0.000541	0.000539	0.000525	0.000540	0.000516	TK
					0.018165	0.018285	0.018130	0.018007	0.017769	
					0.000532	0.000489	0.000479	0.000517	0.000483	
					0.018014	0.017519	0.017345	0.017955	0.017515	
			III	0.000495	0.000524	0.000614	0.000610	0.000575	0.000478	Lindley
				0.017874	0.018073	0.018882	0.019370	0.018901	0.017097	
					0.000518	0.000601	0.000585	0.000542	0.000467	
					0.018046	0.018848	0.018760	0.018152	0.017012	
					0.000548	0.000536	0.000497	0.000550	0.000518	TK
					0.018363	0.018156	0.017500	0.018459	0.018336	
					0.000540	0.000504	0.000477	0.000521	0.000509	
					0.018249	0.017757	0.017168	0.017743	0.017770	
